# Recombinant *Mycobacterium smegmatis* delivering a fusion protein of human macrophage migration inhibitory factor (MIF) and IL-7 exerts an anticancer effect by inducing an immune response against MIF in a tumor-bearing mouse model

**DOI:** 10.1136/jitc-2021-003180

**Published:** 2021-08-13

**Authors:** Hyein Jeong, So-Young Lee, Hyejun Seo, Bum-Joon Kim

**Affiliations:** 1 Department of Microbiology and Immunology, College of Medicine, Seoul National University, Seoul 110799, Korea; 2 Department of Biomedical Sciences, College of Medicine, Seoul National Universtiy, Seoul 03080, Korea; 3 Liver Research Institute, College of Medicine, Seoul National University, Seoul 03080, Korea; 4 Cancer Research Institute, College of Medicine, Seoul National University, Seoul 03080, Korea; 5 Seoul National University Medical Research Center (SNUMRC), Seoul 03080, Korea

**Keywords:** tumor microenvironment, immunotherapy, immunogenicity, vaccine, biomarkers, tumor

## Abstract

**Background:**

Macrophage migration inhibitory factor (MIF) is a pleotropic inflammatory cytokine that is overexpressed in a number of cancer types including most types of human cancer. Inhibition of MIF signaling can restore anticancer immune responses in tumor microenvironments. In this study, we aimed to develop a therapeutic vaccine capable of inhibiting tumor development by inducing anti-MIF immune responses.

**Methods:**

We introduced a recombinant *Mycobacterium smegmatis* (rSmeg-hMIF-hIL-7) vaccine that could deliver a fusion protein of human macrophage migration inhibitory factor (MIF) and interleukin 7, which could act as a target antigen and as an adjuvant of cancer vaccine, respectively. We checked the anticancer potential of the vaccine in a tumor-bearing mouse model.

**Results:**

We found that rSmeg-hMIF-hIL-7 showed enhanced oncolytic activity compared with PBS, BCG or Smeg in MC38-bearing mice, and there was an increase in the humoral and cell-mediated immune responses against MIF. rSmeg-hMIF-hIL-7 can also induce a neutralizing effect regarding MIF tautomerase activity in the serum of vaccinated mice. We also found downregulation of MIF, CD74, and CD44, which are related to the MIF signaling pathway and PI3K/Akt and MMP2/9 signaling, which are regulated by MIF in the tumor tissue of rSmeg-hMIF-hIL-7-vaccinated mice, suggesting a significant role of the anti-MIF immune response to rSmeg-hMIF-hIL-7 in its anticancer effect. In addition, rSmeg-hMIF-hIL-7 treatment led to enhanced activation of CD4^+^ and CD8^+^ T cells in the tumor regions of vaccinated mice, also contributing to the anticancer effect. This trend was also found in LLC-bearing and PanO2-bearing mouse models. In addition, rSmeg-hMIF-hIL-7 treatment exerted an enhanced anticancer effect with one of the immune checkpoint inhibitors, the anti-PD-L1 antibody, in a tumor-bearing mouse model.

**Conclusions:**

In conclusion, our data showed that rSmeg-hMIF-hIL-7 exerts a strong antitumor immune response in mice, possibly by inhibiting the MIF-dependent promotion of tumorigenesis by the anti-MIF immune response and via enhanced cytotoxic T cell recruitment into tumor microenvironments. We also found that it also exerted an enhanced anticancer effect with immune checkpoint inhibitors. These results suggest that rSmeg-hMIF-hIL-7 is a potential adjuvant for cancer immunotherapy. This is the first report to prove anticancer potential of immunotherapeutic vaccine targeting immune response against MIF.

## Background

Cancer immunotherapy is promising as a replacement of other treatments or as adjunctive therapy to enhance the treatment effect of current cancer therapies for a range of different cancer types.^1 2^ Many immunotherapeutic regimens are being approved for the treatment of several types of cancer and are being investigated as immunotherapeutic interventions alone or in combination with conventional treatments.^3–5^

*Mycobacterium smegmatis* (Smeg) is a fast-growing saprophytic environmental mycobacterium^6^ that can be transformed effectively with many heterologous genes, rendering it an ideal vaccine vector. Furthermore, unlike BCG, Smeg is rapidly destroyed by phagolysosomal proteases in the phagosomes of infected cells,^7 8^ which could facilitate better cross-presentation of antigen into T cells. Smeg can also induce the maturation of dendritic cells better than BCG and can trigger CD8^+^ T cell mediated immune responses, suggesting that it has an advantage in inducing the cytotoxic T lymphocyte (CTL) response, which is necessary for cancer immunotherapy.^9 10^ These findings highlight the potential role of Smeg as a recombinant vaccine delivery vector for cancer immunotherapy.

Together with the selection of a proper delivery vector system, immunogenic target antigens for cancer immunotherapy are another crucial option for developing a successful cancer vaccine.^6 10^ Macrophage migration inhibitory factor (MIF) has been characterized as a cytokine implicated in numerous inflammatory diseases, including rheumatoid arthritis, asthma and atherosclerosis.^11–13^ In addition, MIF can also function as a link between inflammation and tumorigenesis.^14^ Recently, MIF expression has been reported to be elevated in various solid tumors with a positive correlation with poor prognosis in patients with cancer.^15 16^

Recently, MIF has been reported to exert its protumorigenic effects through modulation of the immunosuppressive tumor microenvironment, mainly via MIF–CD74 signaling to macrophages and dendritic cells (DCs).^17^ Therefore, inhibiting MIF–CD74 signaling on macrophages and DCs via anti-MIF antibodies or MIF inhibitors could restore the antitumor immune response in tumor microenvironments.^18 19^ In addition, loss of MIF expression in tumor microenvironments can lead to robust induction of a specialized form of cell death, immunogenic cell death (ICD), also contributing to anticancer effects.^20^ These findings highlight the significant role of anti-MIF immune responses or drug inhibitors capable of interfering with MIF signaling in tumor microenvironments in inhibiting tumor development.

Adjuvant interleukin 7 (IL-7) treatment can elicit a significant increase in the number of antigen-specific effector and memory CD8^+^ T cells in several types of vaccine modules, including lentivirus-based or DNA-based vaccines.^21 22^ In particular, IL-7 can boost vaccine-induced antitumor immunity and enhance mouse survival in a tumor-bearing mouse model by enhancing the survival of activated T cells and cytolytic activity in vivo.^23^

Therefore, in this study, to elicit an anti-MIF immune response as an anticancer vaccine, we developed a recombinant Smeg vaccine delivering a fusion protein of human MIF and IL-7 (rSmeg-hMIF-hIL-7), which can act as a target antigen and an adjuvant of cancer vaccine, respectively, via a novel *Mycobacterium–Escherichia coli* shuttle vector system, pMyong2, to guarantee stable and enhanced expression of delivered heterologous genes in recombinant Smeg or BCG.^24 25^ We also sought to explore the immunotherapeutic potential of rSmeg-hMIF-hIL-7 in a tumor-bearing mouse model system. We found that rSmeg-hMIF-hIL-7 treatment led to a significant cancer inhibitory effect mainly by restoring the CTL response in tumor microenvironments by inducing anti-MIF immune responses. In addition, its challenge can exert an enhanced anticancer effect with one of the immune checkpoint inhibitors, anti-PD-L1 antibody, in a tumor-bearing mouse model.

## Materials and methods

### Mice experiments

Female C57BL/6 mice were purchased from Orient Bio and maintained in an ABL-2 laboratory. Seven-week-old C57BL/6 mice were inoculated with MC38, LLC, and PanO2 cells (3×10^6^ cells/mouse) by subcutaneous injection on day 0. For bacterial injection, the mice were treated with peritumoral injections of mycobacterium (2×10^6^ bacteria/mouse) on days 3, 7, and 14. For anti-PD-L1 immunotherapy, the mice were treated by intraperitoneal injection of anti-PD-L1 on days 7 and 14. The tumor size was measured once every 2 or 3 days and calculated following the formula: tumor volume (mm^3^) = (longest diameter × shortest diameter^2^)/2. The mice were observed until the tumor diameter was over 3 mm. Tumor-infiltrating lymphocytes (TILs) were isolated from mice by Ficoll assay^26^ on day 23 and subcutaneously injected into other tumor-bearing mice (3×10^6^ cells/mouse).

### Statistical analysis

Data are shown as the mean±SD and were analyzed by using GraphPad Prism V.9 statistical software (GraphPad, California, USA). Significant differences among multiple groups were analyzed by one-way analysis of variance (ANOVA) followed by Dunnett’s multiple comparison test. A p value of <0.05 was considered to denote statistical significance. *p<0.05, **p<0.01, and ***p<0.001.

## Results

### Construction of recombinant *Mycobacterium smegmatis* expressing the hMIF-hIL-7 fusion protein

**Figure 1:**
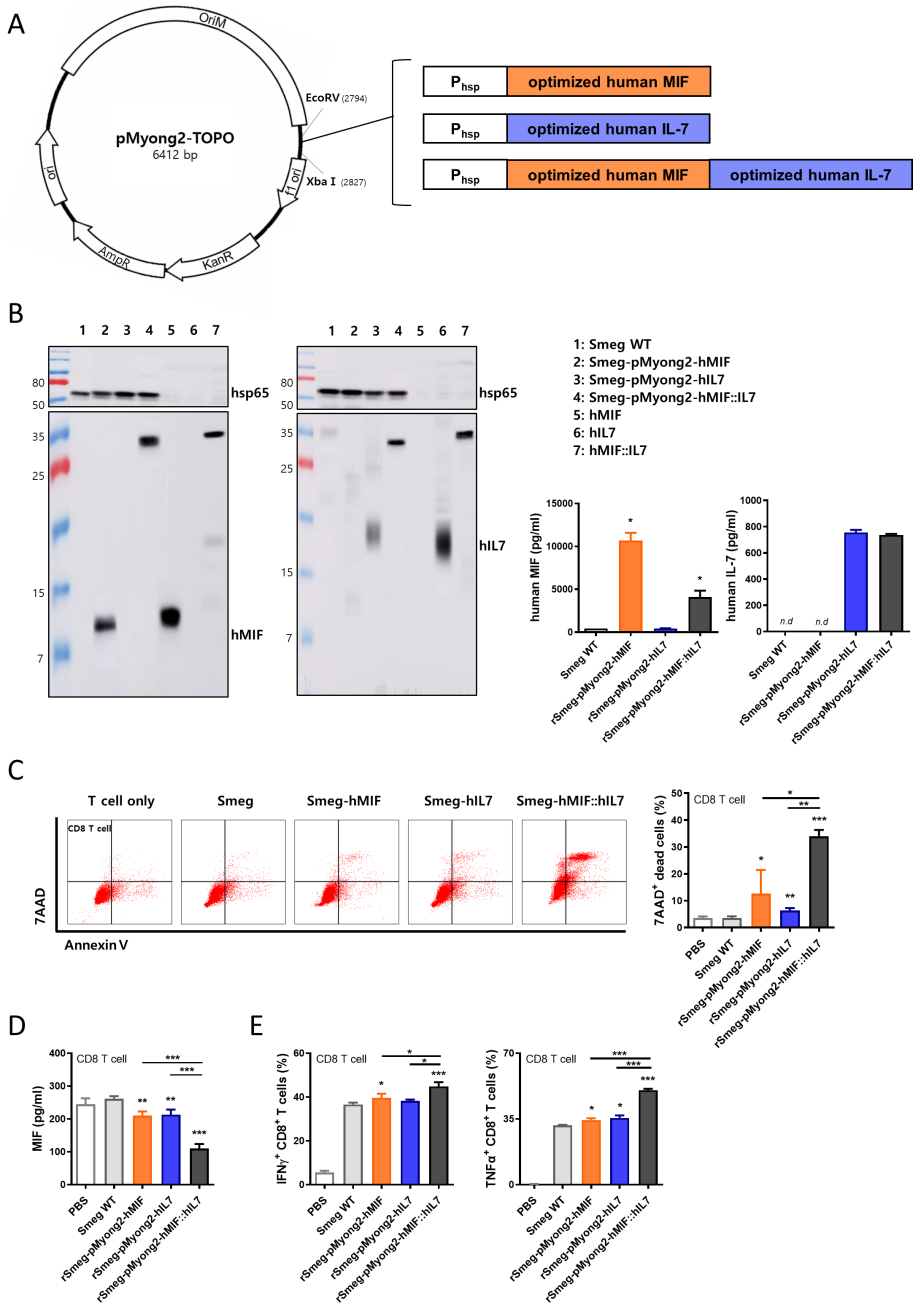
Construction of recombinant *Mycobacterium smegmatis* expressing the hMIF-hIL-7 fusion protein. (A) Maps of a novel *Mycobacterium–Escherichia coli* shuttle vector using pMyong2 expressing the hMIF-hIL-7 fusion protein. (B) Bacteria were cultured until the OD600 value reached 1, and the pellets were sonicated. The bacterial lysates were subjected to SDS-PAGE and analyzed by immunoblot assay. Target protein amounts in the bacterial lysates of rSmeg-hMIF-hIL-7 were assessed by ELISA. (C) For *in vitro* T cell-mediated cytotoxicity assay, MC38 cells were cocultured with CD8^+^ T cells that had been cocultured with bone marrow-derived dendritic cells infected with mycobacteria and analyzed by apoptosis assay. (D) Cell culture supernatants were detected by ELISA. (E) Cytokine-releasing T cell responses in the coculture experiment were analyzed by flow cytometry. Significance differences (*p<0.05, **p<0.01, and ***p<0.001) among the different groups are shown in the related figures, and the data are presented as the means±SEM of four independent experiments.

In a previous study, the protein expression efficacy of the pMyong2 vector system, a novel *Mycobacterium–E. coli* shuttle vector, was found to be significantly higher than the protein expression efficacy of other vector systems.^27^ Using the pMyong2-TOPO vector system, we generated three types of recombinant Smeg strains expressing human macrophage migration inhibitory factor (rSmeg-pMyong2-hMIF), human IL-7 (rSmeg-pMyong2-hIL-7), and fusion protein (rSmeg-pMyong2-hMIF-hIL-7) to enhance the anticancer effect of Smeg^10^ (figure 1A). Protein expression was detected by western blotting assay against human MIF (12 kDa) and human IL-7 (17.5 kDa) after lysis of cultured bacteria. The hMIF-hIL-7 fusion protein expressed in rSmeg-hMIF-hIL-7 was size shifted compared with the single human MIF and human IL-7 proteins. The protein expression levels were also confirmed by ELISA. Human MIF and hMIF-hIL-7 fusion proteins in each bacterial lysate were detected by ELISA against human MIF. In the same way, human IL-7 and hMIF-hIL-7 proteins were detected against IL-7 (figure 1B). Thereafter, to compare the cytotoxic effect induced by mycobacteria, MC38 cancer cells were cocultured with CD8^+^ T cells that had been cocultured with bone marrow-derived DCs infected with various types of rSmeg. There was no difference in T cell activation among the mycobacteria-infected DCs (online supplemental figure S1). However, rSmeg-hMIF-hIL-7-infected dendritic cells significantly induced CD8^+^ T cells capable of killing MC38 cancer cells compared with rSmeg-hMIF or rSmeg-hIL-7, leading to decreased MIF secretion from the cancer cells (figure 1C and D). Interestingly, rSmeg-hMIF-hIL-7-induced CD8^+^ T cells significantly secreted inflammatory cytokines in the presence of MC38 cells (figure 1E). Collectively, these results suggested that CD8^+^ T cells cocultured with DCs infected with rSmeg-hMIF-hIL-7 could exert the strongest CTL response against cancer cells, resulting in reduced MIF secretion from cancer cells.

10.1136/jitc-2021-003180.supp1 Supplementary data

### rSmeg-hMIF-hIL-7 suppressed tumor progression by downregulating MIF and PI3K/Akt signaling

**Figure 2:**
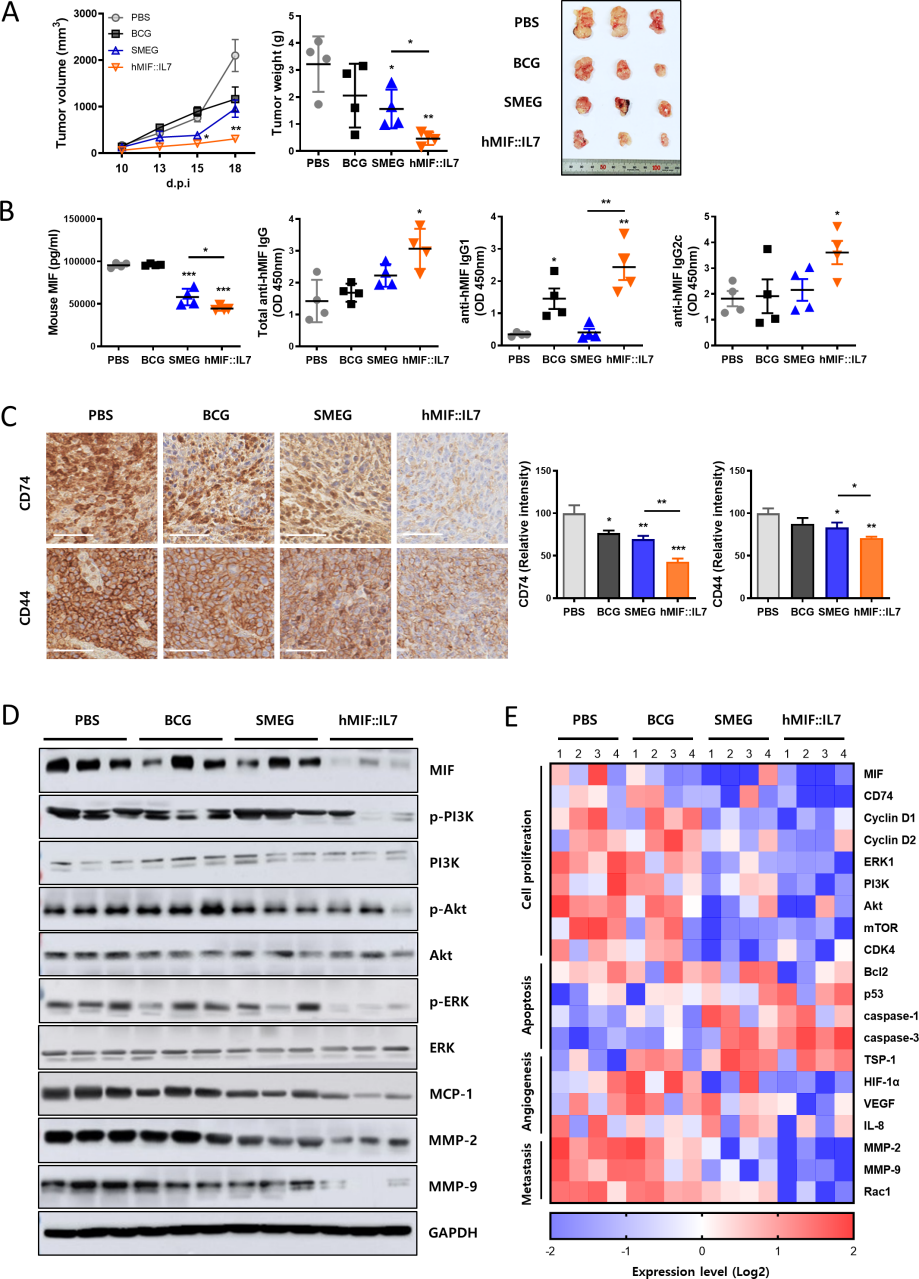
rSmeg-hMIF-hIL-7 suppressed tumor progression by downregulating MIF and PI3K/Akt signaling. C57BL/6 mice were subcutaneously injected with MC38 cells, and mycobacteria were administered on day 3, 7, and 14 after tumor implantation. (A) Growth curve shows tumor volume as the mean±SD at the indicated time points. Representative pictures were taken from tumors of each group, and tumor weight was measured at day 25 post injection (p.i.). (B) Human MIF-specific immunoglobulin subtypes (IgG1, IgG2c, and total IgG) and mouse MIF in serum were assessed by ELISA. (C) Immunohistochemistry staining for the MIF coreceptor CD74 and CD44 (scale bar=50 μm). (D) Protein expression of ERK and PI3K/Akt signaling and MCP-1, matrix metalloproteinases MMP-2 and MMP-9 in primary tumor cells. (E) The transcription level of mRNA in primary tumor cells was quantified by RT-qPCR. Significance differences (*p<0.05, **p<0.01, and ***p<0.001) among the different groups are shown in the related figures, and the data are presented as the mean±SEM of mice (n=4). IL-7, interleukin 7; MIF, macrophage migration inhibitory factor.

Previous studies have shown that recombinant *Mycobacterium smegmatis* has cancer immune therapeutic potential.^6 10^ C57BL/6 mice were subcutaneously inoculated with MC38 colon cancer cells and injected with mycobacteria on days 3, 7, and 14 after cancer injection. In the MC38 tumor-bearing mouse model, rSmeg-hMIF-hIL-7 significantly inhibited tumor progression compared with PBS, BCG, and Smeg. Tumor growth was significantly suppressed by therapeutic administration of rSmeg-hMIF-hIL-7 compared with the other groups (figure 2A). Tumor growth inhibition of rSmeg-hMIF-hIL-7 was also observed in the PanO2-bearing and LLC-bearing mouse model (online supplemental figures S2 and S4). Serum MIF levels, an important modulator in tumor angiogenesis, were decreased in both Smeg and rSmeg-hMIF-hIL-7 compared with PBS, but rSmeg-hMIF-hIL-7-cells showed the most pronounced reduction in MIF levels compared with Smeg. Moreover, administration of rSmeg-hMIF-hIL-7 induced increased production of anti-human MIF IgG1, IgG2c, and total IgG in serum compared with all other groups (figure 2B). The surface expression of MIF coreceptor CD74 and CD44, which promote downstream signaling pathways for cancer cell proliferation and migration,^28^ were decreased in rSmeg-hMIF-hIL-7 compared with all other groups (figure 2C). MIF downstream signaling proteins in primary tumor cells were detected by western blotting. rSmeg-hMIF-hIL-7 administration led to decreased MIF and downstream ERK and PI3K/Akt signaling pathways in primary tumor cells. Also, monocyte chemoattractant protein 1 (MCP-1), one inflammatory cytokine implicated in MDSC tumor infiltration and cancer development, was decreased in response to rSmeg-hMIF-hIL-7. Notably, the production of matrix metalloproteinases MMP-2 and MMP-9, which are crucial for cancer cell invasion and metastasis, was significantly suppressed in response to rSmeg-hMIF-hIL-7 treatment (figure 2D). The transcriptional levels of the corresponding genes were significantly decreased in rSmeg-hMIF-hIL-7 (figure 2E). These results indicate that rSmeg-hMIF-hIL-7 reduced the tumorigenic effect of MIF, enhancing its therapeutic efficacy against tumors.

10.1136/jitc-2021-003180.supp2 Supplementary data

10.1136/jitc-2021-003180.supp3 Supplementary data

### rSmeg-hMIF-hIL-7 induced antitumor immune responses through the recruitment of functional T cells in the tumor environment

**Figure 3:**
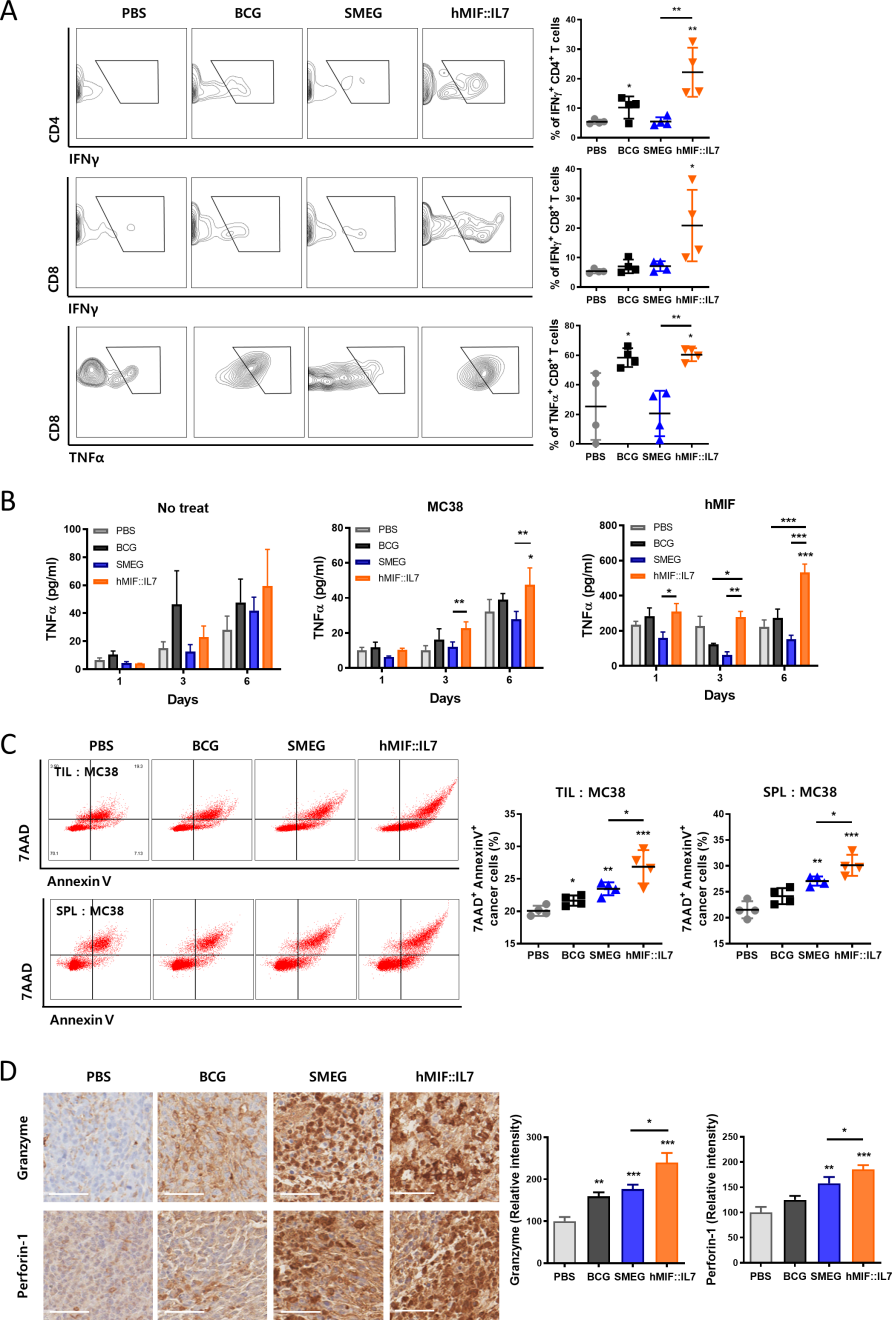
rSmeg-hMIF-hIL-7 induced antitumor immune responses through the recruitment of functional T cells in the tumor environment. (A) The population of IFNγ-releasing CD8^+^ T cells and CD4^+^ T cells and TNFα-releasing CD4^+^ T cells in tumors were analyzed by flow cytometry. (B) Splenocytes were incubated with MC38 lysates or h-MIF for 6 days. Inflammatory cytokine secretion of splenocytes in response to the antigen stimulation was detected by ELISA. (C) Four days after the last mycobacteria injection, lymphocytes from the tumor tissue and spleen were isolated and cocultured with carboxyfluorescein succinimidyl ester (CFSE)-labeled MC38 (E:T=5:1) cancer cells, and cytotoxicity of the lymphocytes were detected by apoptosis assay. (D) IHC image for cytolytic responses by secretion of the pore-forming molecule perforin-1 and proapoptotic protease granzyme B in tumor tissue (scale bar=50 µm). Significance differences (*p<0.05, **p<0.01, and ***p<0.001) among the different groups are shown in the related figures, and the data are presented as the mean±SEM of mice (n=4).

As tumor progression was successfully inhibited through administration of rSmeg-hMIF-hIL-7, a cell-mediated immune response in addition to elevated humoral anti-MIF IgG may work together to inhibit tumor growth. Eleven days after the last mycobacteria injection, rSmeg-hMIF-hIL-7 induced the recruitment of tumor-infiltrating functional T cells. Infiltration of IFNγ-releasing CD8^+^ cytotoxic T cells and CD4^+^ helper T cells in the tumor environment was noticeably induced after rSmeg-hMIF-hIL-7 administration compared with Smeg administration. Additionally, the population of TNFα-releasing CD8^+^ T cells was significantly increased in response to rSmeg-hMIF-hIL-7 compared with Smeg (figure 3A). These enhanced cell-mediated immune responses were also observed in PanO2-bearing and LLC-bearing mouse models (online supplemental figures S3 and S4). In addition, rSmeg-hMIF-hIL-7 significantly enhanced the inflammatory response against MIF in mouse splenocytes compared with Smeg (figure 3B). To assess the cytotoxicity of tumor-infiltrating lymphocytes and splenocytes, lymphocytes were isolated from tumor tissue and spleen and cocultured with MC38 for 24 hours. The 7AAD^+^ AnnexinV^+^ cancer cells were significantly increased by lymphocytes in rSmeg-hMIF-hIL-7 group, indicating that rSmeg-hMIF-hIL-7 administration induced increased lymphocytes capable of directly killing cancer cells in the tumor-bearing mouse (figure 3C). Since interferon-gamma (IFNγ) and tumor necrosis factor-alpha (TNFα) in the tumor environment play a vital role in antitumor activity via the stimulation of tumor-specific cytotoxic T cells,^29^ cytolytic responses by CTLs were also observed by immunohistochemistry (IHC) staining. Secretion of the pore-forming molecule Perforin-1 and proapoptotic protease Granzyme B, which can lead to granule exocytosis of cancer cells, was induced by the administration of rSmeg-hMIF-hIL-7 (figure 3D).

10.1136/jitc-2021-003180.supp4 Supplementary data

### rSmeg-hMIF-hIL-7 inhibited the infiltration of myeloid-derived suppressor cells (MDSCs) into the tumor environment

**Figure 4:**
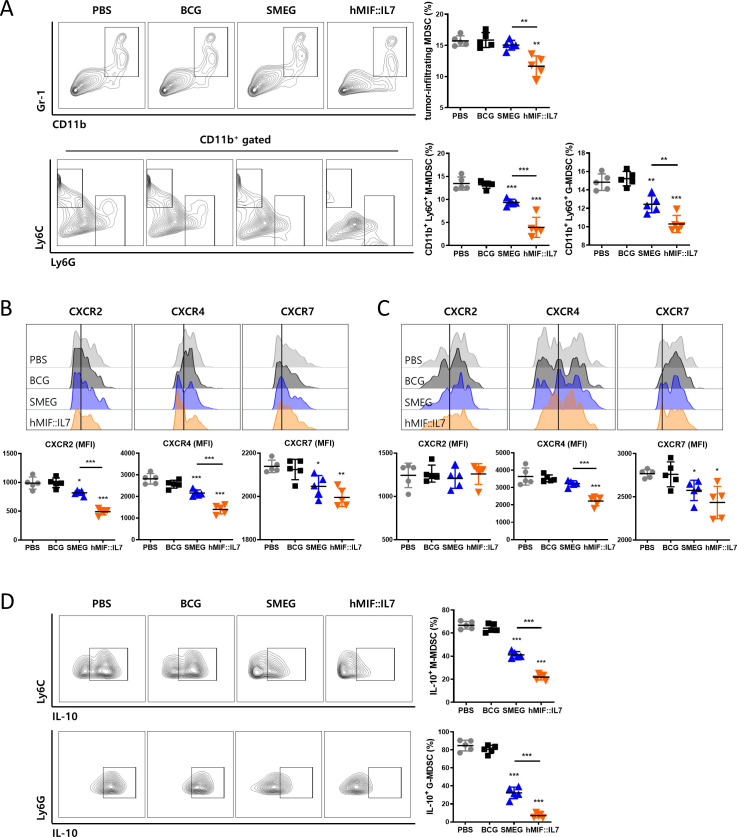
rSmeg-hMIF-hIL-7 inhibited the infiltration of myeloid-derived suppressor cells (MDSCs) into the tumor environment. (A) The population of total MDSC, monocytic MDSC and granulocytic MDSC in mice tumor tissue 4 days after the last mycobacteria injection. Surface expression levels of MIF receptors CXCR2, CXCR4, and CXCR7 of (B) M-MDSC and (C) G-MDSC were detected by flow cytometry. The population of IL-10-releasing (D) M-MDSC and G-MDSC in tumors were analyzed by flow cytometry. Significance differences (*p<0.05, **p<0.01, and ***p<0.001) among the different groups are shown in the related figures, and the data are presented as the mean±SEM of mice (n=5).

As rSmeg-hMIF-hIL-7 administration decreased MIF level in serum and tumor, immune cells population in tumor microenvironment were changed. myeloid-derived suppressor cells (MDSCs), an obstacle to cancer immunotherapies, accumulate in cancer and promotes tumor progression.^30^ Since MDSC accumulation in tumor is dependent on MIF,^31^ MDSC subsets in tumor after rSmeg-hMIF-hIL-7 administration were assessed by flow cytometry. The population of total MDSC, monocytic MDSC (M-MDSC), and granulocytic MDSC (G-MDSC) were significantly decreased in rSmeg-hMIF-hIL-7 group compared with all other group (figure 4A). In addition, among the surface MIF receptors, surface expression of CXCR2 and CXCR4 in M-MDSC, and CXCR4 and CXCR7 in G-MDSC were decreased in response to rSmeg-hMIF-hIL-7, indicating that rSmeg-hMIF-hIL-7 suppressed the infiltration of MDSC into tumor (figure 4B and C). Also, among tumor-infiltrating MDSCs, IL-10-releasing M-MDSC and G-MDSC were decreased in rSmeg-hMIF-hIL-7 compared with all other groups (figure 4D).

### rSmeg-hMIF-hIL-7 inhibited cancer cell proliferation by downregulating the biological activity of serum MIF

**Figure 5:**
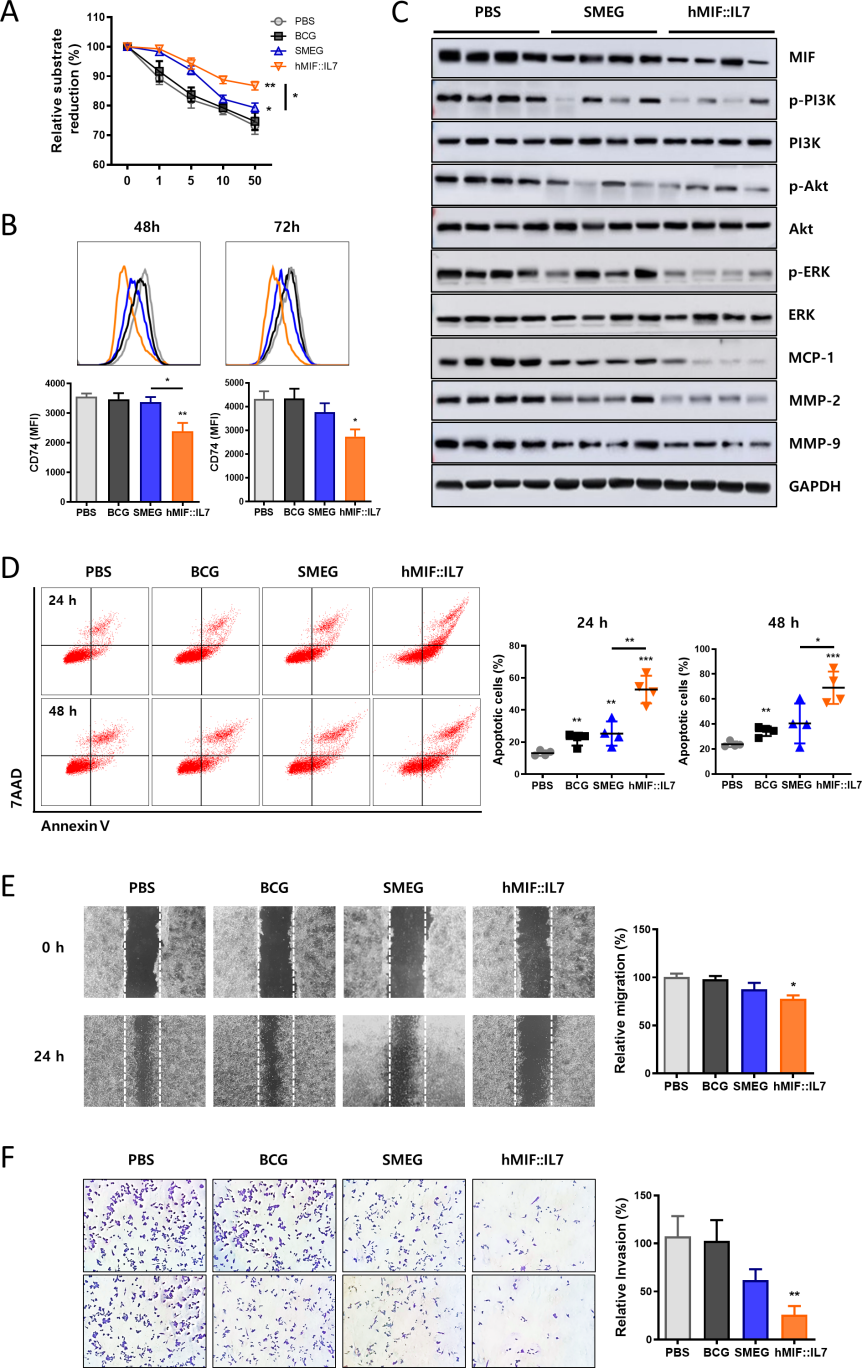
rSmeg-2-hMIF-hIL-7 inhibited cancer cell proliferation by downregulating the biological activity of serum MIF. (A) Tautomerase activity of serum MIF from MC38-bearing mice. (B) Surface expression of CD74 in MC38 cancer cells during incubation with mouse serum. (C) Protein expression of PI3K/Akt signaling and matrix metalloproteinases MMP-2 and MMP-9 in serum-treated MC38 cells. (D) Induction of apoptosis of MC38 cancer cells after 24 h and 48 h incubation in the media containing 50% mouse serum. Inhibition of (E) migration and (F) invasion ability of MC38 cancer cells after incubation with serum from in vivo studies. Significance differences (*p<0.05, **p<0.01, and ***p<0.001) among the different groups are shown in the related figures, and the data are presented as the mean±SEM of mice (n=4). MIF, macrophage migration inhibitory factor.

Since the anti-MIF IgG level in mouse serum was increased in response to rSmeg-hMIF-hIL-7, the serum of vaccinated mice may neutralize MIF and regulate the malignant character of cancer cells. Therefore, next to address this, we checked the biological activity of MIF in vaccinated mice via a tautomerase activity assay. Serum MIF in the rSmeg-hMIF-hIL-7 group showed significantly decreased enzymatic activity compared with the PBS and Smeg groups (figure 5A). Thereafter, MC38 cancer cells showed the decreased expression level of CD74 on the cell surface and downregulated MIF downstream signaling pathway during incubation with serum in rSmeg-hMIF-hIL-7 compared with Smeg, indicating that MIF, which plays a crucial role in cancer cell proliferation, was neutralized by anti-MIF IgG in the serum (figure 5B and C). Serum in the rSmeg-hMIF-hIL-7 and Smeg groups significantly increased the population of apoptotic cancer cells compared with the population of apoptotic cancer cells in the PBS group (figure 5D). Furthermore, the malignant characteristics of cancer cells were suppressed by neutralizing MIF activity. The migration and invasion activities of cancer cells were inhibited after incubation with serum in rSmeg-hMIF-hIL-7 compared with the PBS group (figure 5E and F). Altogether, rSmeg-hMIF-hIL-7 treatment increased neutralization of MIF in serum and suppressed the migration and invasion of cancer cells by neutralizing the biological activity of MIF.

### rSmeg-hMIF-hIL-7 induced tumor-infiltrating lymphocytes capable of downregulating the MIF downstream signaling pathway

**Figure 6:**
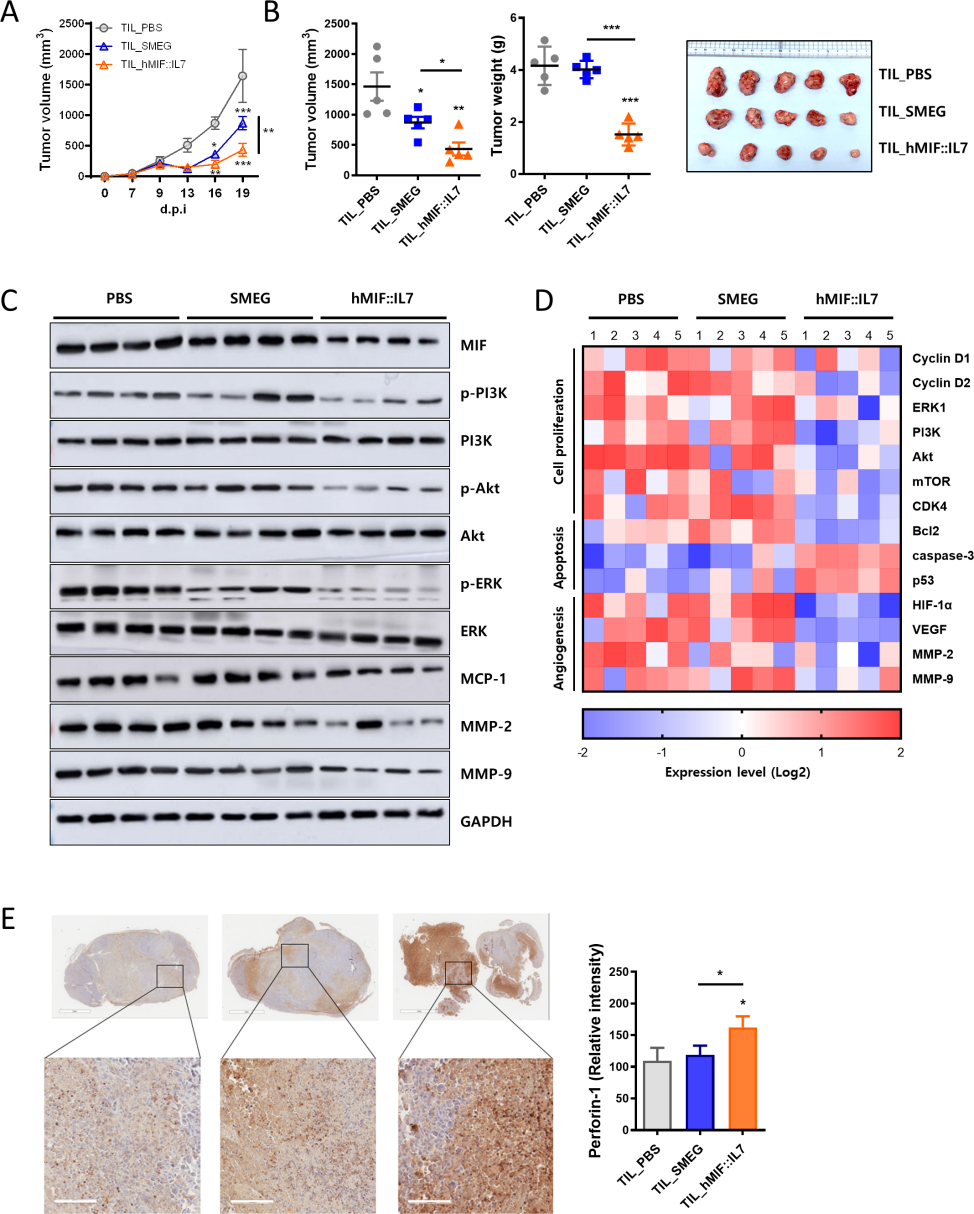
rSmeg-hMIF-hIL-7 induced tumor-infiltrating lymphocytes capable of downregulating the MIF downstream signaling pathway. Tumor-infiltrating lymphocytes from tumor-bearing mice treated with PBS, Smeg, or rSmeg-hMIF-hIL-7 were adoptively transferred into other tumor-established mice. (A) Growth curve represents the tumor volume of tumor-bearing mice as the mean±SD at the indicated time points. (B) Tumor volume and tumor weight were measured at d.p.i. 23. Representative images were taken from tumors of each group at d.p.i. 23. (C) MIF-mediated ERK and PI3K/Akt signaling proteins were detected by western blotting. (D) The transcription level of mRNA in primary tumor cells was quantified by RT-qPCR. (E) Cytolytic protein perforin-1 in tumors was observed by IHC staining (scale bar=100 μm). Significance differences (*p< 0.05, **p<0.01, and ***p<0.001) among the different groups are shown in the related figures, and the data are presented as the mean±SEM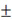 of mice (n=5). MIF, macrophage migration inhibitory factor.

To further confirm the role of tumor-infiltrating lymphocytes in rSmeg-hMIF-hIL-7-mediated function, the anticancer effect of TILs isolated from rSmeg-hMIF-hIL-7-injected mice was observed by adoptively transferring TILs into tumor-bearing recipient mice. TILs from rSmeg-hMIF-hIL-7 treatment significantly suppressed tumor volume compared with those from Smeg treatment (figure 6A and B). Tumor weight at day post injection (d.p.i.) 23 was reduced only in the rSmeg-hMIF-hIL-7 group, not in the PBS and Smeg groups, suggesting that rSmeg-hMIF-hIL-7 induced TILs capable of inhibiting tumor growth in a way that is distinct from Smeg (figure 6B). Notably, protein expression in the ERK and PI3K/Akt downstream pathway of MIF was significantly inhibited by treatment with TILs from the rSmeg-hMIF-hIL-7 group compared with the Smeg group (figure 6C). Moreover, the transcription levels of the corresponding genes were downregulated by treatment with TILs from rSmeg-hMIF-hIL-7 (figure 6D). The cytolytic protein perforin-1 was significantly increased by TILs from rSmeg-hMIF-hIL-7 compared with the TILs from Smeg, suggesting that TILs in response to rSmeg-hMIF-hIL-7 more effectively induced tumor cell death (figure 6E). Interestingly, tumor infiltration of MDSC and surface MIF receptors in MDSCs were decreased in response to treatment with TILs from rSmeg-hMIF-hIL-7 (online supplemental figure S5). Collectively, rSmeg-hMIF-hIL-7 enhanced cell-mediated immune responses against MIF and regulated the MIF-mediated PI3K/Akt pathway in the tumor environment via functional TILs.

10.1136/jitc-2021-003180.supp5 Supplementary data

### rSmeg-hMIF-hIL-7 exerted an enhanced anticancer effect with anti-PD-L1 immunotherapy

**Figure 7:**
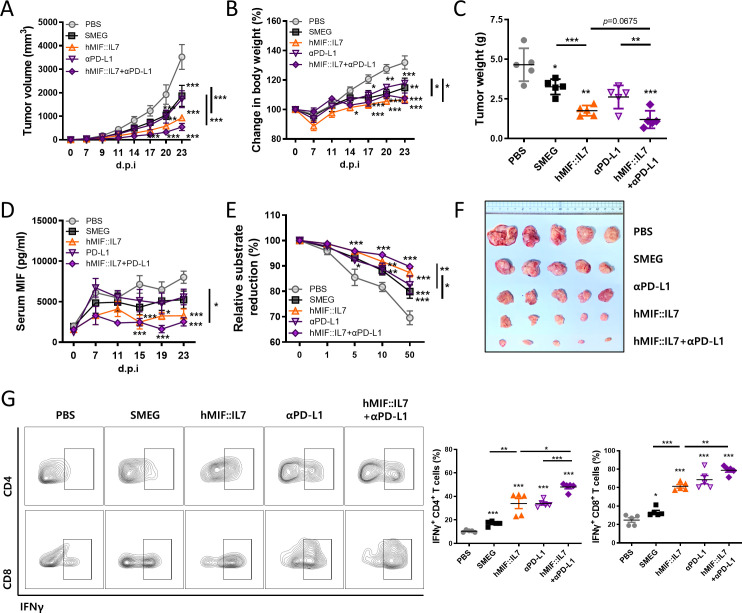
rSmeg-hMIF-hIL-7 exerted an enhanced anticancer effect with anti-PD-L1 immunotherapy. C57BL/6 mice were subcutaneously injected with MC38 cells, anti-PD-L1 was administered by the intraperitoneal route on d.p.i. 7 and 14, and mycobacteria were injected on d.p.i. 3, 7, and 14. Growth curve shows (A) tumor volume and (B) body weight change of tumor-bearing mice as the mean±SD at the indicated time points. (C) The tumor weight was measured at d.p.i. 23. (F) Representative pictures were taken from tumors and spleens of each group at d.p.i. 23. (D and E) Serum MIF and tautomerase activity of serum MIF were detected by ELISA. (G) The population of functional tumor-infiltrating lymphocytes. Significance differences (*p<0.05, **p<0.01, and ***p<0.001) among the different groups are shown in the related figures, and the data are presented as the mean±SEM of mice (n=5). MIF, macrophage migration inhibitory factor.

Next, we checked the enhanced effect of the combination treatment of rSmeg-hMIF-hIL-7 and anti-PD-L1. Our data showed that combination treatment with the immunotherapeutic agents rSmeg-hMIF-hIL-7 and anti-PD-L1 exerted an enhanced anticancer effect. rSmeg-hMIF-hIL-7 alone suppressed tumor growth and body weight change compared with PBS treatment, but the combinational treatment of rSmeg-hMIF-hIL-7 and anti-PD-L1 showed significant reduction in tumor volume and body weight change compared with rSmeg-hMIF-hIL-7 treatment alone or anti-PD-L1 alone (figure 7A and B). Excised tumor weight and size at d.p.i. 23 were drastically decreased by the combination therapy compared with rSmeg-hMIF-hIL-7 alone or anti-PD-L1 alone (figure 7C and F and online supplemental figure S6A). Interestingly, the combinational therapy resulted in the highest spleen/body weight ratio and increased population of cytokine-releasing T cells in the spleen, suggesting that the combination therapy could promote the recruitment of functional immune cells in the spleen (online supplemental figure S6A, E). rSmeg-hMIF-hIL-7 and anti-PD-L1 worked together to induce the recruitment of cytokine-releasing T cells in tumor tissue and suppress tumor-infiltration of MDSCs (figure 7G, online supplemental figures S6D, S7, S8). Notably, the combinational treatment of rSmeg-hMIF-hIL-7 and anti-PD-L1 resulted in decreased serum MIF and biological activity of serum MIF compared with rSmeg-hMIF-hIL-7, indicating that anti-PD-L1 treatment maximizes the ability of rSmeg-hMIF-hIL-7 to neutralize MIF (figure 7D and E and online supplemental figure S6B). These results indicated that rSmeg-hMIF-hIL-7 exerted an enhanced anticancer effect with cancer immune checkpoint drugs, suggesting the potential use of rSmeg-hMIF-hIL-7 as an adjunctive immunotherapy capable of enhancing the effect of immunotherapy.

10.1136/jitc-2021-003180.supp6 Supplementary data

10.1136/jitc-2021-003180.supp7 Supplementary data

10.1136/jitc-2021-003180.supp8 Supplementary data

## Discussion

In the present study, we developed a recombinant Smeg strain, rSmeg-hMIF-hIL-7 as a new anticancer therapeutic vaccine, expressing the chimeric fusion protein of h-MIF as the target Ag of the cancer vaccine and h-IL-7 as an adjuvant of the cancer vaccine to induce anti-MIF immune responses (graphical abstract). We also proved that rSmeg-hMIF-hIL-7 can exert strong anticancer immune responses in various tumor-bearing mouse models (MC38, PanO2 and LLC), which was mediated mainly by eliciting anti-MIF immune responses.

MIF, a member of the tautomerase family of cytokines, is highly expressed in numerous cancers, including colon cancer, lung cancer, breast cancer, pancreatic cancer, melanoma, nasopharyngeal carcinoma, cervical adenocarcinoma, and prostate cancer.^28^ MIF is now recognized as a pleiotropic cytokine that plays pivotal roles in cancer promotion and the inflammatory cascade, and MIF is constitutively expressed in almost all cancer cell types.^32^ MIF can exert various biological activities, including cancer-promoting effects, mainly via its interaction with the receptor complex of the cognitive receptor CD74 and coreceptor CD44. Signaling by binding of MIF with the receptor complex can lead to a cancer-promoting effect mainly by inducing the PI3K/Akt axis,^33^ p53 inhibition by sustained ERK activation^34^ or enhanced angiogenesis following VEGF and MMP activation by upregulation of HIF-1. In addition to cancer cells, it has been proven that MIF could affect innate myeloid, neutrophil and adaptive Th1, Th2, and NK T cells, influencing tumor-associated immune responses.^35–37^ MIF promotes the tumor infiltration and immune suppressive activity of MDSCs in mouse models of breast, lung, and glioblastoma cancer.^31 35^ Also, MIF induces Th2 and Th17 expansion and suppresses CTL activation, which are primary target of immune checkpoint inhibitor immunotherapies.^37^ Moreover, since the expression of MIF has been reported to be positively correlated with the severity of the cancer phenotype,^38^ MIF is an attractive target for the development of cancer therapeutic inhibitors.

Currently, many efforts have focused on the development of small molecule inhibitors that target the unique tautomerase active site of MIF and anti-MIF antibodies capable of neutralizing MIF. For example, ISO-1 treatment capable of inhibiting MIF tautomerase activity has been reported to markedly inhibit the tumorigenic growth of various cancers, including prostate cancer, colon cancer and pancreatic cancers, by effectively blocking the interaction between MIF and receptors, particularly CD74,^32^ leading us to hypothesize that an immunotherapeutic vaccine approach eliciting an anti-MIF immune response can also play a pivotal role in cancer treatment. To date, an immunotherapeutic vaccine targeting MIF for cancer treatment has not been introduced. Theoretically, immunotherapeutic vaccines have merit over chemical inhibitors or anti-MIF antibodies since the former can also lead to a cell-mediated immune response against MIF-producing cancer cells in addition to a humoral immune response against MIF capable of neutralizing MIF activity.

Since MIF has various biological functions, including inflammation induction or cancer promotion,^32^ direct MIF protein challenge as a vaccine component could have a risk of generating unwanted biological phenotypes. Therefore, to use MIF as a vaccine, its accurate loading to antigen-presenting cells (APCs) such as macrophages or DCs is an essential step. For this reason, we chose *M. smegmatis* for vaccine delivery of MIF in this study, which generally infects APCs, and loaded antigens can easily be processed and presented into CD4^+^ or CD8^+^ T cells, resulting in strong immune responses against delivered Ags. Importantly, since it leads to an enhanced CTL response against cancer and grows more rapidly than BCG,^10^ it seems more appropriate for the delivery of cancer vaccines in terms of treatment efficacy or production cost.

IL-7 has various vaccine potentiating effects, including differentiating naïve T cells into memory T cells or activating γδ T cells. Therefore, we generated rSmeg strains expressing fusion proteins of both hMIF and hIL-7 to maximize anti-MIF immune responses in vaccinated mice. Indeed, we found that rSmeg-hMIF-hIL-7 elicited an enhanced CTL response against CD4^+^ and CD8^+^ T cells cocultured with infected dendritic cells compared with rSmeg expressing h-MIF alone or h-IL-7 alone (figure 1), suggesting an adjuvant effect of IL-7 in cancer immunotherapy.

Our data indicated that the anticancer effect of rSmeg-hMIF-hIL-7 in tumor-bearing mice could be mediated mainly by two major mechanisms: attenuation of MIF signaling by interaction with CD74, perhaps via anti-MIF antibody-mediated reduction of MIF levels or neutralization of MIF activity, and attenuation of the CD4^+^ or CD8^+^ T cell mediated immune response against MIF, particularly the CD8^+^ T cell mediated CTL response in tumor microenvironments. Our data indicated that rSmeg-hMIF-hIL-7 can lead to an enhanced humoral immune response against MIF (an increase in anti-MIF IgG capable of neutralizing tautomerase activity in serum) in vaccinated mice (figures 2 and 3). Given that MIF tautomerase activity is positively correlated with MIF cancer-promoting signaling,^39^ an increase in anti-MIF IgG induced by rSmeg-hMIF-hIL-7 vaccination is likely to elicit anticancer effects by blocking MIF signaling. It has been reported that MIF could also regulate expression of receptor complex, CD74 and CD44,^40–42^ resulting in affecting MIF signaling. Indeed, we found that MIF, CD74, and CD44, which are related to the MIF signaling pathway, and PI3K/Akt and MMP2/9 signaling-mediated regulation MIF were downregulated in the tumor tissue of rSmeg-hMIF-hIL-7-vaccinated mice (figure 2), suggesting a significant role of the anti-MIF humoral immune response by rSmeg-hMIF-hIL-7 in its anticancer effect. In addition to the anti-MIF humoral immune response, a reduction in the entire secreted MIF level in serum or tumor microenvironments could also contribute to the anticancer effect via robust induction of a specialized form of cell death, immunogenic cell death (ICD),^20^ and inhibition of MIF signaling. Our data showed that T cells cocultured with dendritic cells that had been infected with rSmeg-hMIF-hIL-7 could reduce secreted MIF levels via enhanced killing of MIF-producing cancer cells (figure 1), suggesting that the reduced MIF level found in mice vaccinated with rSmeg-hMIF-hIL-7 may be due to dual actions of anti-MIF antibodies and an enhanced CTL response.

T lymphocytes play a key role in electing antitumor immunity and are the major targets of immune checkpoint inhibitor (ICI) immunotherapies. Tumor-derived MIF can compromise T lymphocyte mediated anticancer effects, particularly the CTL response in tumor microenvironments.^43^ Therefore, efforts to reduce MIF levels in tumor microenvironments using anti-MIF antibodies or small inhibitors have been reported to be able to restore MHC I expression on tumors to allow for more effective CTL-mediated immunotherapy in tumor microenvironments.^44^ Indeed, our data indicated that the anticancer potential of CTLs from the tumor tissue of rSmeg-hMIF-hIL-7-vaccinated mice was enhanced compared with the anticancer potential of CTLs from control mice (figure 3), suggesting that the reduction in tumor-derived MIF levels by rSmeg-hMIF-hIL-7 vaccination can contribute to anticancer effects by recovering CTL function in the tumor microenvironment in vaccinated mice. Moreover, we found that adoptive transfer of isolated TILs from rSmeg-hMIF-hIL-7-injected mice led to a strong anticancer effect in a tumor-bearing mouse model via inhibition of MIF signaling (figure 5), indicating that cell-mediated immune responses against MIF could play a major role in the anticancer effect. Of note, our immunotherapeutic approach using rSmeg-hMIF-hIL-7 showed an enhanced anticancer effect with one of the ICI drugs, anti-PD-L1 treatment, in a tumor-bearing mouse model (figure 6), suggesting its further feasibility in cancer treatment.

Of the potential small molecule inhibitors of MIF signaling, ibudilast has gained a great attention due to its strong anti-inflammatory effect and suppressive effect of MDSCs by MCP-1 inhibition via blocking MIF signaling.^45^ Of note, it can lead to the most powerful reduction of MDSC generation compared with other MIF inhibitors 4-IPP and ISO-1.^45^ Ibudilast can also contribute to cancer treatment via increasing CD8^+^ T cell infiltration and suppressing MDSC functions in tumor microenvironments^45 46^ in a MIF-dependent manner. As shown in ibudilast treatment, our data also indicated that rSmeg-hMIF-hIL7 can decrease population of total MDSC, M-MDSC, and G-MDSC in tumor microenvironments of vaccinated mice (figure 4), possibly due to of its inhibition of MIF signaling, suggesting that it can contribute to recovery of T cell-mediated anticancer function in tumor microenvironments via MDSC suppression. In T cell-mediated anticancer effect, rSmeg-hMIF-hIL7 may have merit over ibudilast in that the former can lead to vaccine-mediated activation of T lymphocytes capable of killing MIF-producing cancer cells as well as recovery of T cell function mediated by MDSC inhibition.

The present study has several limitations. Our results include rSmeg-hMIF-hIL7 anticancer therapy only in female mice. However, there exists gender disparity in immune responses.^47^ So, rSmeg-hMIF-hIL7 anticancer effect should also be evaluated in male mice in the future. Furthermore, synergistic effect of rSmeg-hMIF-hIL7 anticancer therapy with other MIF inhibitors such as ISO-1 or Ibudilast remains to be evaluated in the future.

In conclusion, our results demonstrate that rSmeg-hMIF-hIL-7 therapy elicits a strong anticancer immune response in mice, mainly via reduction of tumor-derived MIF level, neutralization of MIF or the recruitment of activated lymphocytes capable of downregulating the MIF downstream signaling pathway, indicating the potential use of rSmeg-hMIF-hIL-7 as adjunctive immunotherapy capable of enhancing the efficacy of immunotherapy.

10.1136/jitc-2021-003180.supp9 Supplementary data

10.1136/jitc-2021-003180.supp10 Supplementary data

10.1136/jitc-2021-003180.supp11 Supplementary data

## Data Availability

All data relevant to the study are included in the article or uploaded as supplementary information. The original contributions presented in the study are included in the article/supplementary material; further inquiries can be directed to the corresponding author.
