# Supplementary figures and images for: Recombinant Mycobacterium smegmatis delivering a fusion protein of human macrophage migration inhibitory factor (MIF) and IL-7 exerts an anticancer effect by inducing an immune response against MIF in a tumor-bearing mouse model
Source: J Immunother Cancer. 2021 Aug 13;9(8):e003180. doi: 10.1136/jitc-2021-003180 (PMC8365831; doi:10.1136/jitc-2021-003180)

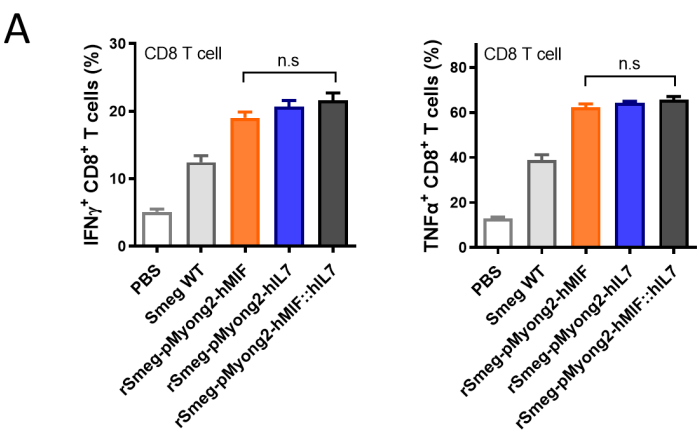

Supplement: Supplementary data [file jitc-2021-003180supp001.pdf]

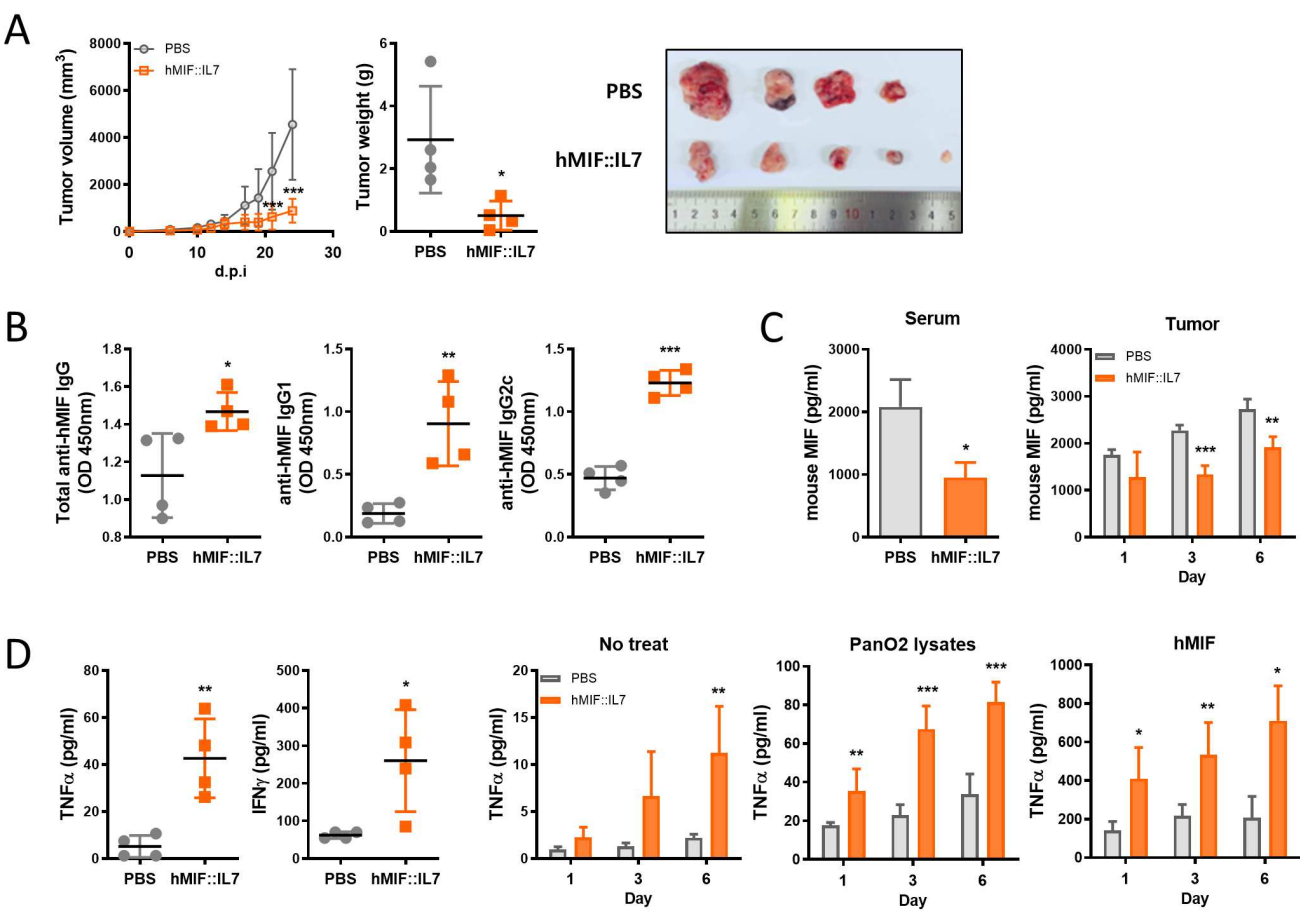

Supplement: Supplementary data [file jitc-2021-003180supp002.pdf]

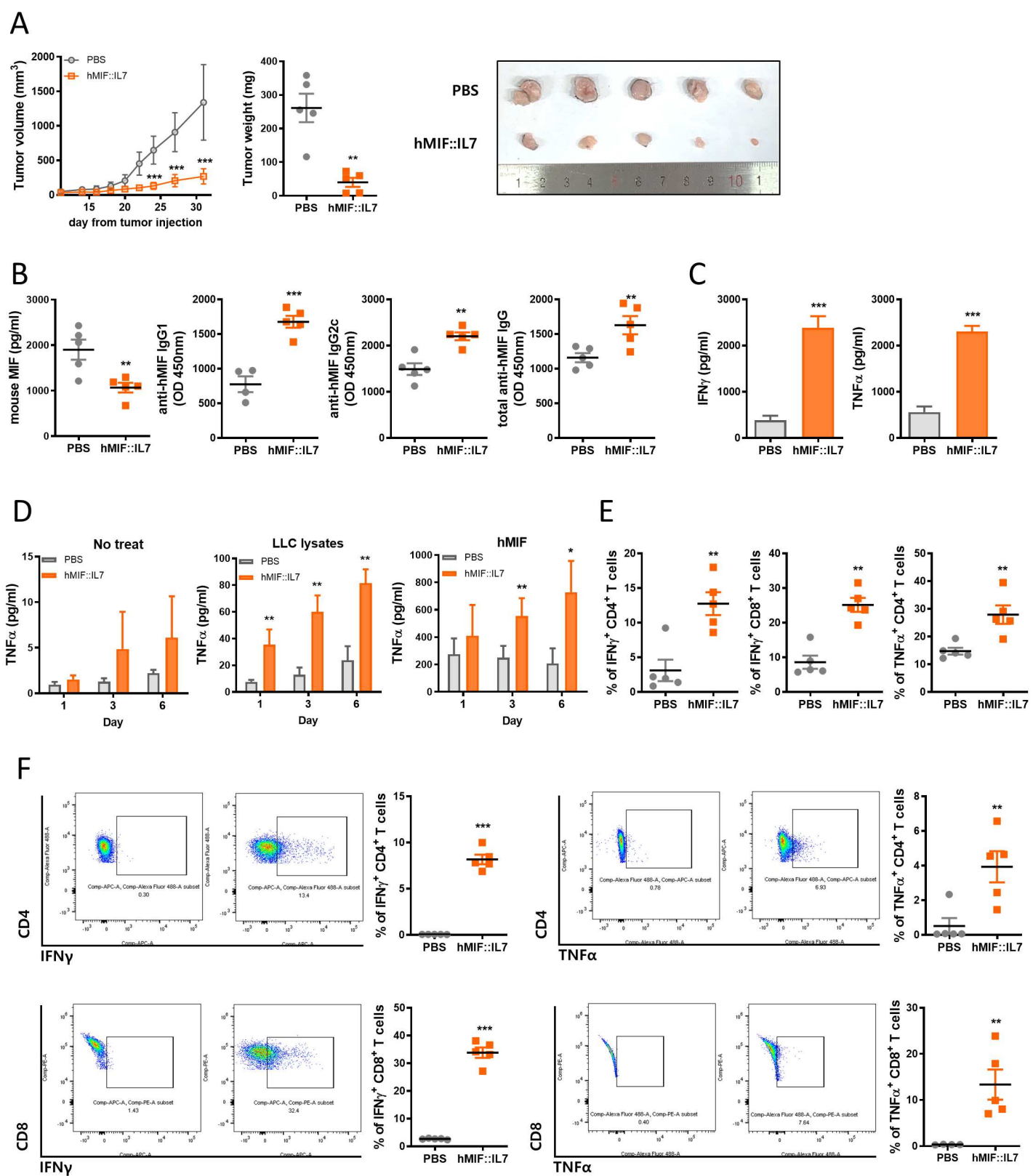

Supplement: Supplementary data [file jitc-2021-003180supp003.pdf]

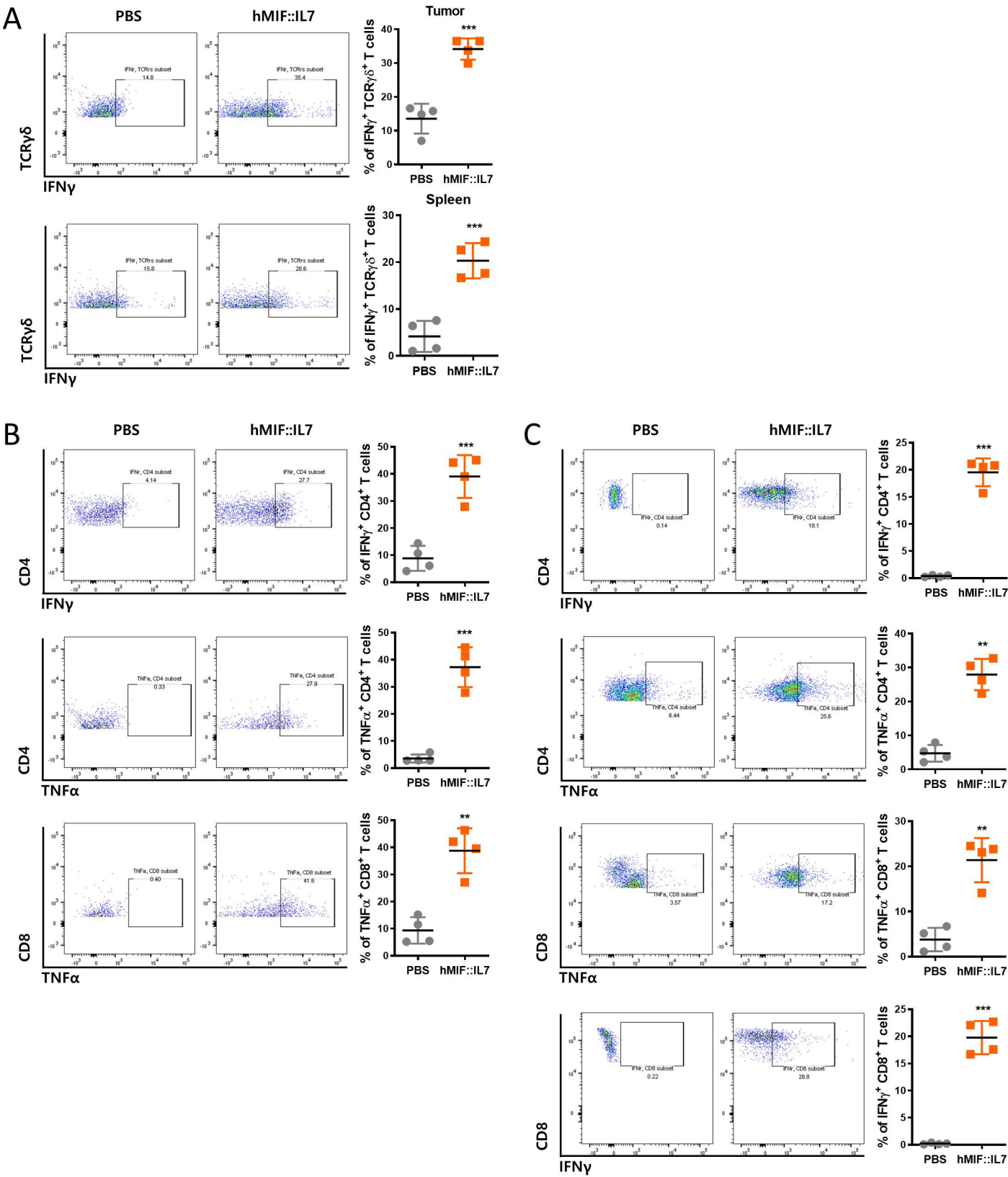

Supplement: Supplementary data [file jitc-2021-003180supp004.pdf]

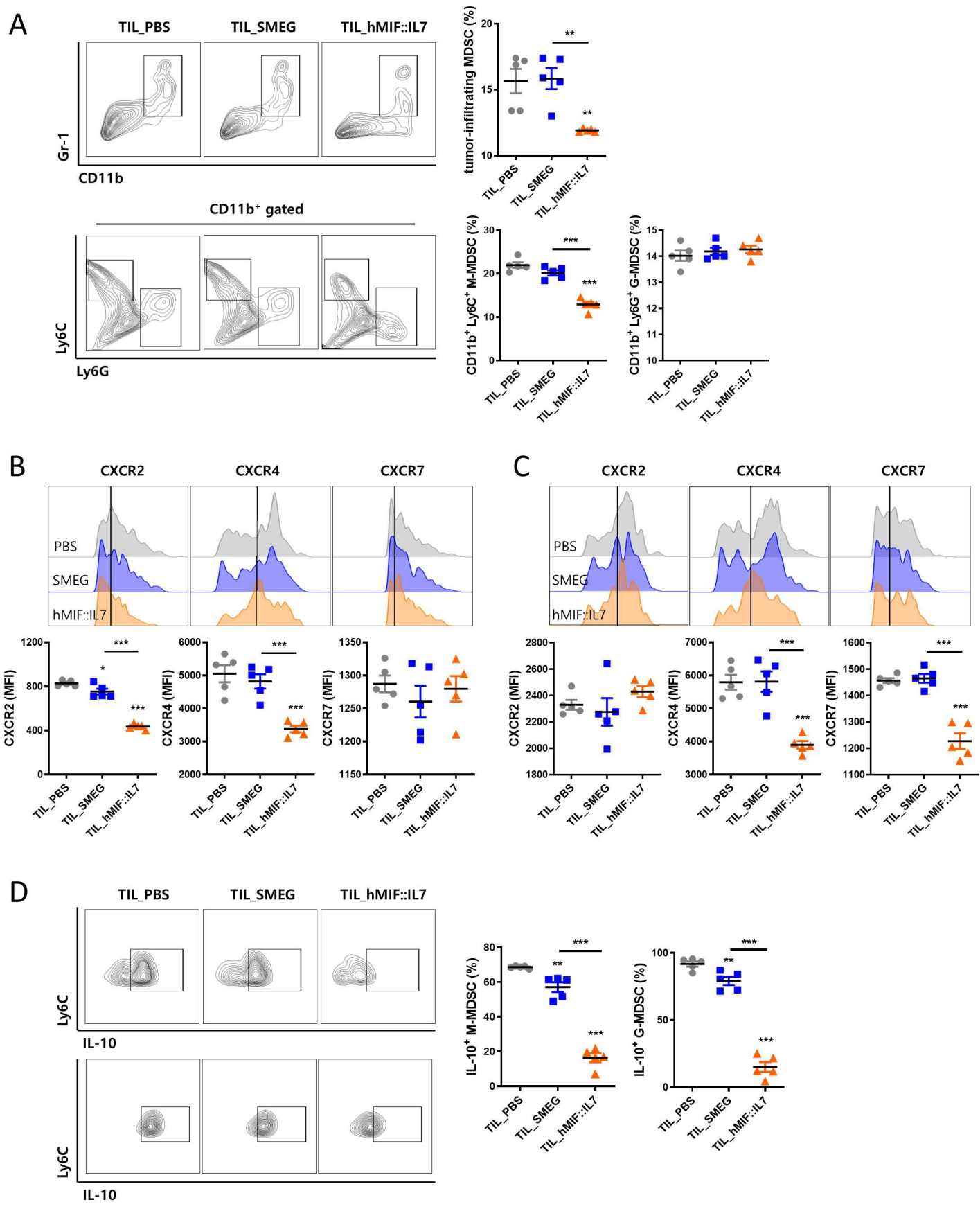

Supplement: Supplementary data [file jitc-2021-003180supp005.pdf]

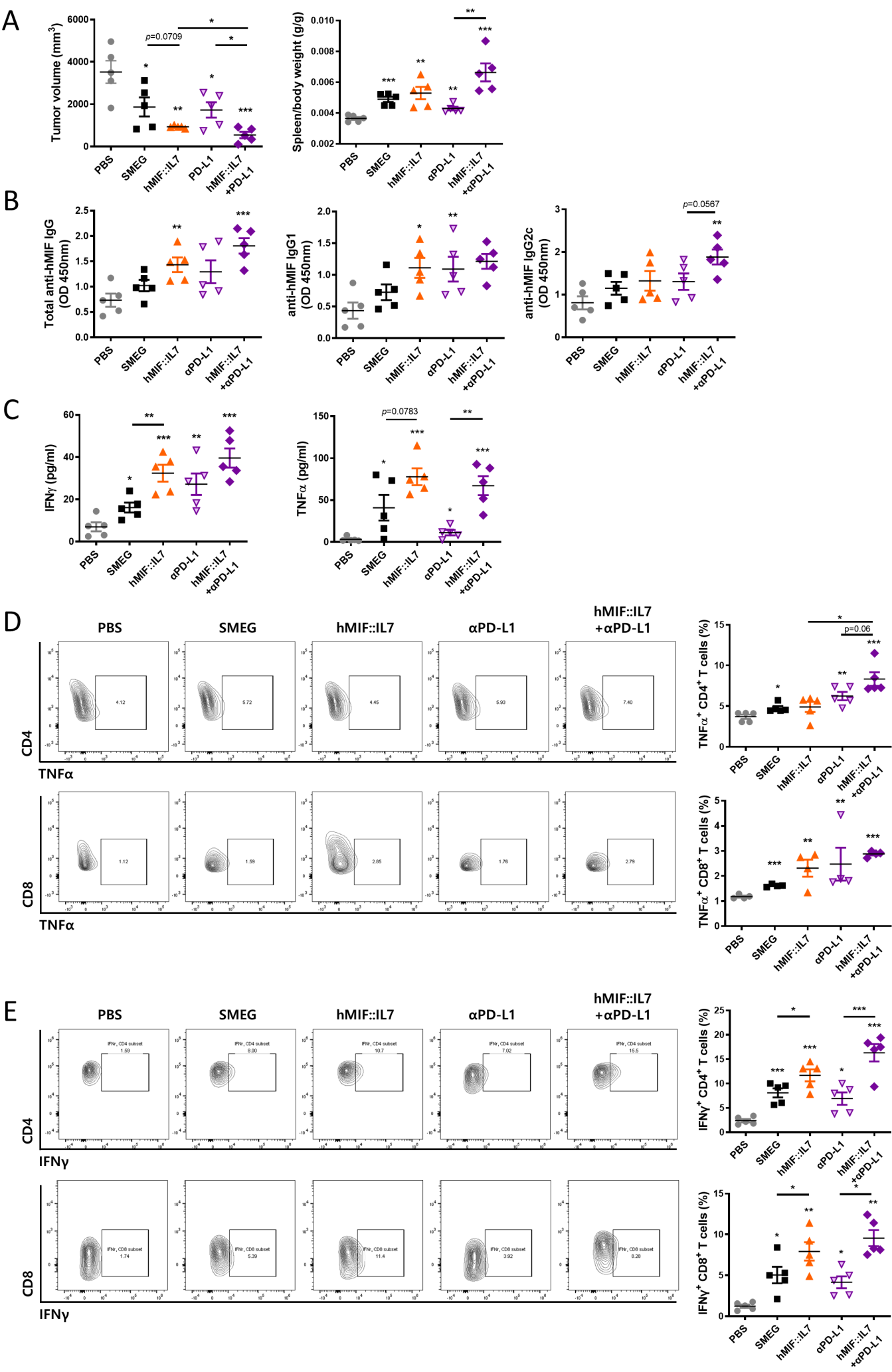

Supplement: Supplementary data [file jitc-2021-003180supp006.pdf]

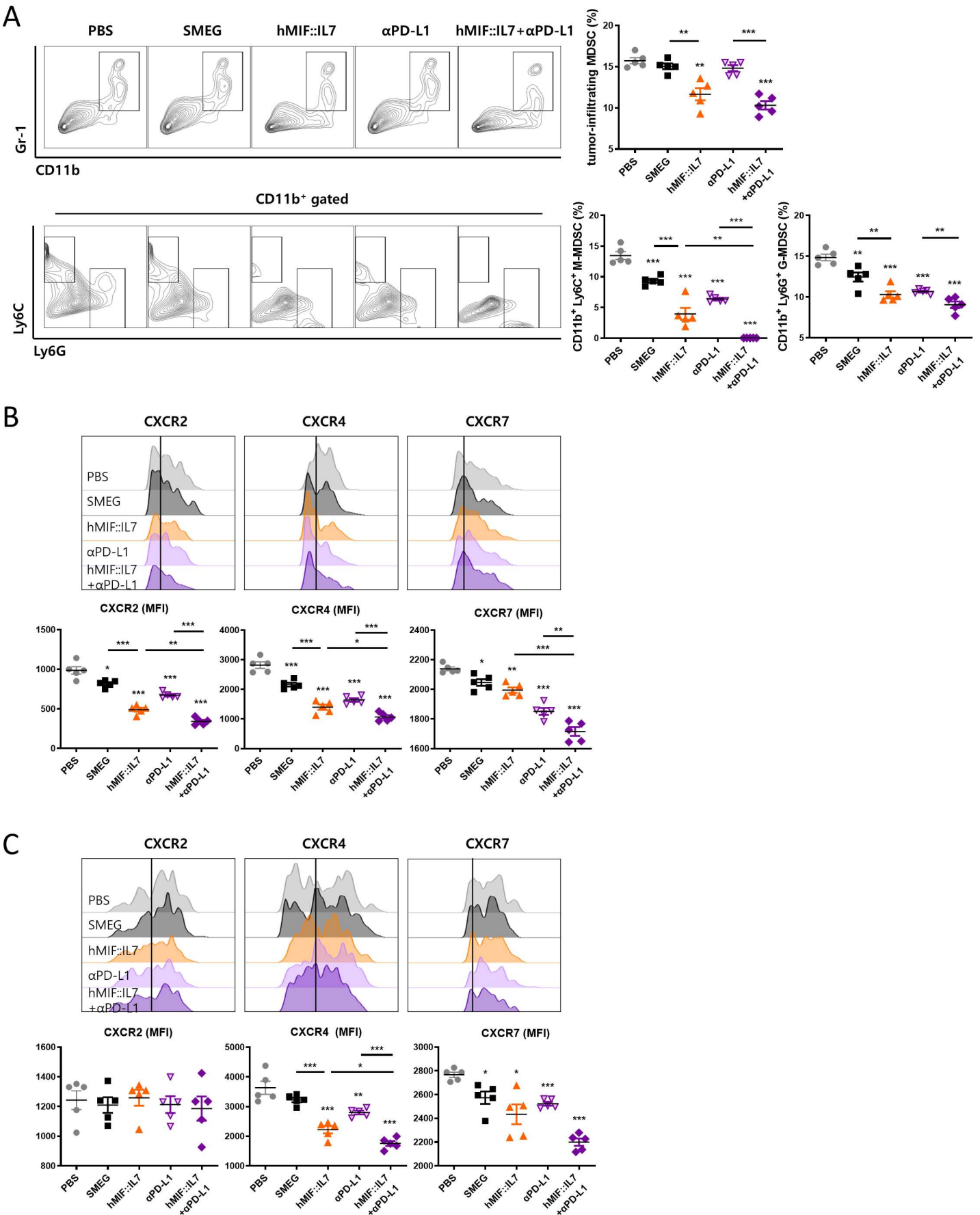

Supplement: Supplementary data [file jitc-2021-003180supp007.pdf]

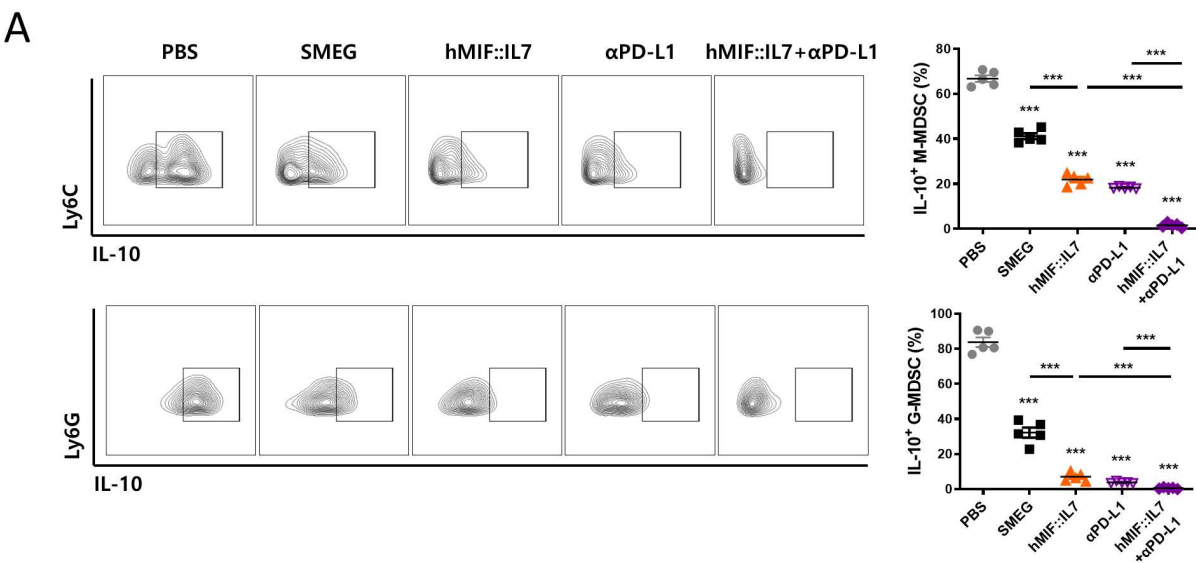

Supplement: Supplementary data [file jitc-2021-003180supp008.pdf]
