# Supplementary material for: Recombinant Mycobacterium smegmatis delivering a fusion protein of human macrophage migration inhibitory factor (MIF) and IL-7 exerts an anticancer effect by inducing an immune response against MIF in a tumor-bearing mouse model
Source: J Immunother Cancer. 2021 Aug 13;9(8):e003180. doi: 10.1136/jitc-2021-003180 (PMC8365831; doi:10.1136/jitc-2021-003180)
Supplement: Supplementary data [file jitc-2021-003180supp009.pdf]

Supplementary materials

Materials and Methods

Construction of recombinant *Mycobacterium smegmatis* expressing human MIF, human IL-7, or fusion protein hMIF-hIL-7

For human MIF, human IL-7, and fusion protein to be smoothly expressed in *M. smegmatis*, codon optimization was carried out using the JCat tool (**48**). The codon-optimized DNA sequences were synthesized by the gene synthesis service of Bionics (KR). *MIF* and *IL7* were amplified in the synthesized oligonucleotide using combinations of primers. For effective expression in *Mycobacterium*, a promoter region of *hsp65* (P_{hsp65}) was amplified in *M. bovis* BCG and conjugated by overlap PCR so that the insert was located downstream of the promoter. To make *MIF*:*IL7* downstream of P_{hsp65}, amplified P_{hsp65}-*MIF* and IL-7 were reamplified with other combinations of primers and conjugated by overlap PCR (**Supplementary Table S1**). The constructed inserts and pMyong2-TOPO vector, *Mycobacterium*-*E. coli* shuttle vectors introduced in a previous study were digested by EcoRV-HF and XbaI (NEB, USA), ligated by a DNA ligation kit (TaKaRa, JP), and transformed into the *E. coli* DH5α strain (RBC, Taiwan). After preparing the plasmid using the NucleBond Xtra Maxi Plus kit (Macherey-Nagel, DE), 2 μg of each plasmid was mixed with 200 μL of competent *M. smegmatis* mc^2 155, washed three times with ice-cold 10% glycerol and finally resuspended in 5 ml of ice-cold 10% glycerol. The mix was transferred onto 0.2 cm-gap electroporation cuvettes (Bio-Rad, USA) and electroporated for 2.5 μF, 1000 Ω, and 2500 V using Gene Pulser Xcell™ (Bio-Rad). The electroporated *M. smegmatis* was recovered in 5 ml of 7H9 broth for 4 h at 37°C and spread with proper dilution onto 7H10 with 100 μg/ml kanamycin.

Cell culture

The mouse colon cancer cell line MC38 and mouse pancreatic cancer cell line PanO2 were grown in complete Dulbecco's modified Eagle medium (DMEM, Life Technologies, CA, USA) containing 10% FBS and 100 U/ml penicillin/streptomycin in a humid environment containing CO_2 and air at 37°C. The mouse lung cancer cell line LLC was grown in complete RPMI 1640 (Life Technologies, CA, USA) supplemented with 10% FBS and 100 U/ml penicillin/streptomycin. Splenocytes isolated from tumor-bearing mice were incubated in complete RPMI 1640 supplemented with IL-2 for 6 days with cancer cell lysates (3:1 cancer cell equivalents) or 5 µg/ml of h-MIF.

Bacterial growth conditions

Escherichia coli strain DH5α (RBC) used in plasmid construction was grown in Luria-Bertani broth with shaking incubation or on LB agar overnight at 37°C. To select the transformed *E. coli*, 100 µg/ml kanamycin was used. *Mycobacterium smegmatis* mc^2 155 was grown in Middlebrook 7H9 broth (BD Biosciences, USA) supplemented with 10% ADC (BD Biosciences, USA), 2.5% glycerol, and 0.2% Tween-80 or on 7H10 plates supplemented with 10% OADC (BD Biosciences, USA) and 0.5% glycerol. To select transformed *M. smegmatis*, 100 µg/ml kanamycin was used.

Flow cytometry

Tumor tissues from tumor-bearing mice were dissociated with collagenase IV (0.5 mg/ml, Sigma, USA) and DNase I (20 µg/ml, Sigma, USA). Single tumor cells and splenocytes mashed through the cell strainer were incubated with mAbs, including anti-CD8, anti-CD4, anti-TCRγδ and anti-CD3 antibodies, for 30 min on ice. For intracellular cytokine staining of

IFNγ and TNFα, cells were cultured for 4 h with PMA (50 ng/ml) and ionomycin (1 μg/ml), and cytokine release was prevented by treatment with brefeldin A. Following fixation/permeabilization, cells were stained with mAbs against IFNγ and TNFα. All antibodies were purchased from BD Biosciences (USA). Fluorescence was measured by using FACSForteessa (BD Biosciences, USA) and FlowJo software (BD Biosciences, USA).

Wound healing assay

Cells were seeded and allowed to grow to 95% confluency in a CO_2 incubator. Wounds were introduced to a monolayer of cells using a sterile 200-μl pipette tip. The cells were incubated in media containing 50% mouse serum for 24 h. Then, the wounded monolayer of cells was washed with PBS and cultured. The speed of wound closure was observed and calculated by using ImageJ.

Invasion assay

Cell invasion was detected by using a Transwell assay with a pore size of 8 μm coated with Matrigel (Corning, USA). Cancer cells were resuspended in media containing 20% mouse serum, and the cell density was adjusted to 2×10^5 cells/200 μl in the upper well of the Transwell chamber. The lower compartment was filled with 500 μl of 40% FBS medium. After 24 h incubation, the cells in the upper well were wiped by using a wet cotton swab. Traversed cells on the lower side of the filter were fixed in methanol for 30 min and then stained with Hoechst (Sigma, USA) for 30 min. The cells were counted microscopically and quantified by ImageJ.

***invitro* T cell-mediated cytotoxicity assay**

Naïve $CD8^+$ T cells were isolated from mouse spleen and cocultured for 96 h with bone-

marrow derived dendritic cells that had been infected with mycobacteria at a DC:T cell ratio of 1:10. Thereafter, MC38 cells were cocultured with $CD8^+$ T cells for 48 h at a T cell:MC38 ratio of 5:1, resuspended in binding buffer and stained with 7AAD-PE-Cy5 and Annexin V-PE on ice according to the manufacturer's guidelines for apoptosis detection kits (BD Biosciences, USA). Flow cytometry was performed by a FACSFortessa (BD Biosciences, USA). The data were analyzed by FlowJo software (BD Biosciences, USA).

Histopathological study

For histological analysis, the fixed tumor tissues were embedded in paraffin and sectioned. Each sectioned slide was stained with hematoxylin and eosin (H&E), and immunohistochemistry for CD74, CD44, Granzyme B, and Perforin-1 (all antibodies purchased from Santa Cruz) was performed. Overall staining intensity was analyzed using ImageJ.

MIF tautomerase activity assay

The tautomerase activity of MIF in the serum was detected as previously described (**49**). MIF converts phenylpyruvate from the enol- to the keto- form, and this reaction was detected by the decreased absorbance at 280 nm on a spectrophotometer. The assay mixture contained a series of diluted MIF-containing serum samples and 40 mM sodium phosphate buffer (pH 6.5) samples. Ethanol-diluted phenylpyruvate was added to the assay mixture at a final concentration of 100 μM, and the absorbance values were monitored using a Tecan microplate reader (CH).

Cell apoptosis assay

Cell apoptosis was assessed by using flow cytometry. MC38 cells were incubated in media

supplemented with 50% mouse serum for 24 h and 48 h, resuspended in binding buffer and stained with 5 µl 7AAD-PE-Cy5 and 2 µl Annexin V-PE for 15 min on ice according to the manufacturer's guidelines in apoptosis detection kits (BD Biosciences, USA). Flow cytometry was assessed using a FACSFortessa (BD Biosciences, USA) and were analyzed by FlowJo software (BD Biosciences, USA).

ELISA

ELISA plates (Corning, USA) were coated with 5 µg/ml of human MIF protein in PBS overnight at 4°C. Mouse serum was diluted and added to each well. Following incubation with HRP-conjugated anti-mouse IgG, IgG1, and IgG2c antibodies, the plates were developed with 3,3', 5,5'-tetramethylbenzidine. The reactions were stopped with 1 N hydrochloric acid, and the absorbance was measured at 450 nm using a Tecan microplate reader (CH). The concentration of cytokines in mouse serum was measured with an ELISA kit purchased from Biolegend (USA) for human MIF, Abcam (ENG) for human IL-7, R&D Systems (USA) for mouse MIF, and Invitrogen (USA) for mouse TNF, IL-6, and IFNγ, according to the manufacturer's procedure.

Western blot assay

Tumor tissues were lysed in a radio immunoprecipitation assay (RIPA, Thermo Fisher Scientific, USA) containing protease inhibitor cocktail and phosphatase inhibitor and homogenized. The proteins were collected and quantified using the Bradford assay (Bio-Rad, USA). For Western blotting assays, 100 µg protein samples were boiled for 5 min at 95°C, separated on a 10% SDS-PAGE gel and transferred onto a nitrocellulose membrane. Then, the membranes were blocked in 5% BSA for 1 h at room temperature. Primary monoclonal antibodies (all from Cell Signaling Tech, USA) were incubated with the membrane overnight

at 4°C. After repeated washing, the membranes were incubated with the corresponding horseradish peroxidase-labeled secondary antibodies for 2 h. The protein blots were examined by using enhanced chemiluminescence (ECL) reagents (Bio-Rad, USA).

RNA extraction and RT-qPCR

Total mRNA was extracted from the tumor tissue using TRIzol reagent (Invitrogen, USA), quantified, and transcribed into complementary DNA by reverse transcriptase. The transcription level of target genes was detected using a CFX Connect real-time system (Bio-Rad, USA) and analyzed by RT-qPCR with sets of primers (**Supplementary Table S2**), and the housekeeping gene GAPDH was used as an internal control. The fold change was calculated using the delta delta CT method (**50**) and normalized by the average delta CT value of all groups.

Figure titles and legends

Supplementary Table S1. Primer sets used in rSmeg-hMIF-hIL-7 construction.

Supplementary Table S2. Primer sets used for RT-qPCR.

Supplementary Figure S1. Cytokine response of CD8 T cells cocultured with bone marrow-derived dendritic cells that had been infected with mycobacteria. Significance differences (*$p < 0.05$, **$p < 0.01$, ***$p < 0.001$) among the different groups are shown in the related figures, and the data are presented as the mean ± s.e.m. of four independent experiments.

Supplementary Figure S2. Anticancer effects of rSmeg-hMIF-hIL-7 in a PanO2 tumor-

bearing mouse model. (**A**) PanO2 tumor volume was decreased after injection with rSmeg-hMIF-hIL-7 on d.p.i. 3, 7, and 14. (**B**) Antihuman MIF IgG levels in serum. (**C**) MIF levels in serum and culture medium of primary tumor cells. (**D**) Cytokines in serum and splenocytes from tumor-bearing mice stimulated with PanO2 lysates and human MIF. Significance differences (**p* < 0.05, ***p* < 0.01, ****p* < 0.001) among the different groups are shown in the related figures, and the data are presented as the mean ± s.e.m. of mice (n=4).

Supplementary Figure S3. rSmeg-hMIF-hIL-7 inhibits tumor progression through the recruitment of functional T cells in the PanO2 tumor environment. (**A**) Accumulation of TCRγδ T cells in tumor tissue and spleen. (**B**) Infiltration of cytokine-releasing immune cells into tumor tissue. (**C**) Accumulation of activated immune cells in the spleen. Significance differences (**p* < 0.05, ***p* < 0.01, ****p* < 0.001) among the different groups are shown in the related figures, and the data are presented as the mean ± s.e.m. of mice (n=4).

Supplementary Figure S4. rSmeg-hMIF-hIL-7 inhibits tumor progression in an LLC tumor-bearing mouse model. (**A**) LLC tumor size was decreased after rSmeg-hMIF-hIL-7 treatments. (**B**) MIF amounts and anti-MIF IgG levels in serum from LLC tumor-bearing mice. (**C**) Systemically induced cytokine production in serum. (**D**) Inflammatory cytokines after antigen restimulation. (**E**) Functional tumor-infiltrating lymphocytes. (**F**) Cytokine-releasing lymphocytes in spleen. Significance differences (**p* < 0.05, ***p* < 0.01, ****p* < 0.001) among the different groups are shown in the related figures, and the data are presented as the mean ± s.e.m. of mice (n=5).

Supplementary Figure S5. Tumor-infiltrating lymphocytes from tumor-bearing mouse after injection with rSmeg-hMIF-hIL-7 inhibited tumor-infiltration of myeloid-derived suppressor cells (MDSCs). MC38 tumor-bearing mouse were injected two times with mycobacteria (2×10^6 bacteria/mouse) on d.p.i 3, 7, and tumor-infiltrating lymphocytes were transferred two days after the last injection to TIL-recipent tumor-bearing mouse. (A) The population of total and monocytic MDSC and granulocytic MDSC in tumor tissue at d.p.i 16. (B) Surface MIF receptors CXCR2, CXCR4, and CXCR7 in MDSC, and (C) cytokine-releasing MDSCs were assessed by flow cytometry. Significance differences (*$p < 0.05$, **$p < 0.01$, ***$p < 0.001$) among the different groups are shown in the related figures, and the data are presented as the mean ± s.e.m. of mice (n=5).

Supplementary Figure S6. rSmeg-hMIF-hIL-7 exerted an enhanced anticancer effects with antiPD-L1 immunotherapy. (**A**) Anti-human MIF IgG levels in serum five days after the last mycobacteria or anti-PD-L1 injection. (**B**) Systemically induced cytokine production in serum five days after the last mycobacteria or anti-PD-L1 injection. The population of functional immune cells in (**C**) tumor and (**D**) spleen. Significance differences (*$p < 0.05$, **$p < 0.01$, ***$p < 0.001$) among the different groups are shown in the related figures, and the data are presented as the mean ± s.e.m. of mice (n=4~5).

Supplementary Figure S7. Combination therapy of rSmeg-hMIF-hIL-7 and anti-PD-L1 exerted an significant inhibition effect of tumor-infiltration of MDSCs. MDSC subsets were assessed four days after the last mycobacteria injection. (**A**) The population of total MDSC, M-MDSC, and G-MDSC, and the expression level of surface MIF receptors in (**B**)

BMJ Publishing Group Limited (BMJ) disclaims all liability and responsibility arising from any reliance placed on this supplemental material which has been supplied by the author(s)

M-MDSC and (**C**) G-MDSC were assessed by flow cytometry. Significance differences (**p* < 0.05, ***p* < 0.01, ****p* < 0.001) among the different groups are shown in the related figures, and the data are presented as the mean ± s.e.m. of mice (n=5).

Supplementary Figure S8. Combination therapy of rSmeg-hMIF-hIL-7 and anti-PD-L1 exerted an significant inhibition effect of cytokine-releasing MDSCs. MDSC subsets were assessed four days after the last mycobacteria injection. The population of IL-10-releasing (**A**) M-MDSC and G-MDSC in tumor were assessed using flow cytometry. Significance differences (**p* < 0.05, ***p* < 0.01, ****p* < 0.001) among the different groups are shown in the related figures, and the data are presented as the mean ± s.e.m. of mice (n=5).
