# Supplementary material for: Recombinant Mycobacterium smegmatis delivering a fusion protein of human macrophage migration inhibitory factor (MIF) and IL-7 exerts an anticancer effect by inducing an immune response against MIF in a tumor-bearing mouse model
Source: J Immunother Cancer. 2021 Aug 13;9(8):e003180. doi: 10.1136/jitc-2021-003180 (PMC8365831; doi:10.1136/jitc-2021-003180)
Supplement: Supplementary data [file jitc-2021-003180supp010.pdf]

Supplementary table S1

Target sequence	Primer name	Sequence (5'-3')
**P_{hsp65} (for MIF)**	EcoRV_Phsp_F	TTTGATATCGGTGACCACAACGACGCGC (EcoRV)
	Phsp_m_R	GTTGGTGTTCACGATGAACATCGGCATTGCGAAGTGATTCCTCCGGATCG
**MIF**	p_op.hMIF_F	CGATCCGGAGGAATCACTTCGCAATGCCGATGTTCATCGTGAACACCAAC
	op.hMIF_XbaI_R	AAATCTAGATTAGGCGAAGGTCGAGTTGTTCCA (XbaI)
**P_{hsp65}-MIF**	EcoRV_Phsp_F	TTTGATATCGGTGACCACAACGACGCGC (EcoRV)
	op.hMIF_XbaI_R	AAATCTAGATTAGGCGAAGGTCGAGTTGTTCCA (XbaI)
**P_{hsp65} (for IL7)**	EcoRV_Phsp_F	TTTGATATCGGTGACCACAACGACGCGC (EcoRV)
	Phsp_7_R	CCCTCGATGTCGCAGTCCATTGCGAAGTGATTCCTCCGGATCG
**IL7**	p_op.hIL7_F	CGATCCGGAGGAATCACTTCGCAATGGACTGCGACATCGAGGG
	op.hIL7_XbaI_R	AAATCTAGATTAGTGCTCCTTGGTGCCCATCAGGAT (XbaI)
**P_{hsp65}-IL7**	EcoRV_Phsp_F	TTTGATATCGGTGACCACAACGACGCGC (EcoRV)
	op.hIL7_XbaI_R	AAATCTAGATTAGTGCTCCTTGGTGCCCATCAGGAT (XbaI)
**P_{hsp65}-MIF (for P_{hsp65}-MIF:IL7)**	EcoRV_Phsp_F	TTTGATATCGGTGACCACAACGACGCGC (EcoRV)
	op.hMIF_7_R	TGCCCTCGATGTCGCAGTCCATGGCGAAGGTCGAGTTGTTCCAG
**IL7 (for P_{hsp65}-MIF:IL7)**	m_op.hIL7_F	CTGGAACAACTCGACCTTCGCCATGGACTGCGACATCGAGGGCA
	op.hIL7_XbaI_R	AAATCTAGATTAGTGCTCCTTGGTGCCCATCAGGAT (XbaI)
**P_{hsp65}-MIF:IL7**	EcoRV_Phsp_F	TTTGATATCGGTGACCACAACGACGCGC (EcoRV)
	op.hIL7_XbaI_R	AAATCTAGATTAGTGCTCCTTGGTGCCCATCAGGAT (XbaI)

Supplementary table S2

Target sequence	Foward primer (5'-3')	Reverse primer (5'-3')
**MIF**	CGTGCCGCTAAAAGTCATGA	GCAAGCCCGCACAGTACAT
**CD74**	ATGACCCAGGACCATGTGATG	CCCTTCAGCTGCGGGTACT
**Cyclin D1**	GCGTACCCTGACACCAATCTC	CTCCTCTTCGCACTTCTGCTC
**Cyclin D2**	GAGTGGGAACTGGTAGTGTTG	CGCACAGAGCGATGAAGGT
**Cdk4**	ATGGCTGCCACTCGATATGAA	TCCTCCATTAGGAACTCTCACAC
**mTOR**	CTGGGACTCAAATGTGTGCAGTTC	GAACAATAGGGTGAATGATCCGGG
**ERK1**	GCTCGACCACACTGGCTTTC	GATCAACTCCTTCAGCCGCTC
**PI3K**	AGGAGCGGTACAGCAAAGAA	GCCGAACACCTTTTTGAGTC
**Akt**	TGAAAACCTTCTGTGGGACC	TGGTCCTGGTTGTAGAAGGG
**caspase-3**	GCTGGACTGCGGTATTGAGA	CCATGACCCGTCCCTTGA
**caspase-1**	ACAAGGCACGGGACCTATG	TCCCAGTCAGTCCTGGAAATG
**Bcl2**	TGAGTACCTGAACCGGCATCT	GCATCCCAGCCTCCGTTAT
**BAX**	TGAAGACAGGGGCCTTTTTG	AATTCGCCGGAGACACTCG
**BAD**	GCCCTAGGCTTGAGGAAGTC	CAAACTCTGGGATCTGGAACA
**p53**	CACGTACTCTCCTCCCCTCAAT	AACTGCACAGGGCACGTCTT
**TSP-1**	GCAGCACACACAGAAGCATT	CAATCAGCTCTCACCAGCAG
**HIF-1a**	GAAATGGCCCAGTGAGAAAA	CTTCCACGTTGCTGACTTGA
**VEGF**	TGCTCACTTCCAGAAACACG	GGAAGGGTAAGCCACTCACA
**IL-8**	ATGGCTGCTCAAGGCTGGTC	AGGCTTTTCATGCTCAACACTAT
**MMP-2**	GAGTTGGCAGTGCAATACCT	GCCGTCCTTCTCAAAGTTGT
**MMP-9**	AGTTTGGTGTCGCGGAGCAC	TACATGAGCGCTTCCGGCAC
**Rac1**	GAGACGGAGCTGTTGGTAAAA	ATAGGCCCAGATTCACTGGTT
**GAPDH**	TGGATTTGGACGCATTGGTC	TTGCACTGGTACGTGTTGAT
