# Supplementary material for: Recombinant Mycobacterium smegmatis delivering a fusion protein of human macrophage migration inhibitory factor (MIF) and IL-7 exerts an anticancer effect by inducing an immune response against MIF in a tumor-bearing mouse model
Source: J Immunother Cancer. 2021 Aug 13;9(8):e003180. doi: 10.1136/jitc-2021-003180 (PMC8365831; doi:10.1136/jitc-2021-003180)
Supplement: Supplementary data [file jitc-2021-003180supp011.pdf]

Recombinant *Mycobacterium smegmatis* delivering a fusion protein of human macrophage migration inhibitory factor (MIF) and IL-7 exerts an anticancer effect by inducing an immune response against MIF in a tumor-bearing mouse model

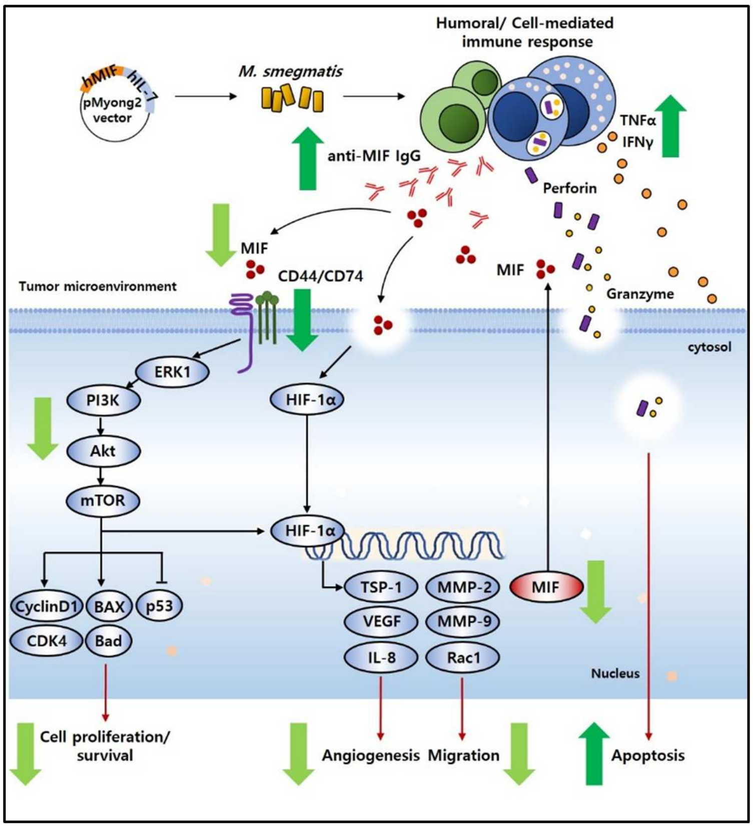

Authors
Hyein Jeong, So-Young Lee, Hyejun Seo, and Bum-Joon Kim

Correspondence
kbumjoon@snu.ac.kr

In brief

We developed recombinant *Mycobacterium smegmatis* expressing human MIF and IL-7 (rSmeg-hMIF-hIL-7) to function as a cancer vaccine targeting MIF. It can exert an anticancer effect via induction of humoral and cell-mediated immune response against MIF in tumor microenvironments of vaccinated mice.
